# Exosome-Derived MicroRNAs of Human Milk and Their Effects on Infant Health and Development

**DOI:** 10.3390/biom11060851

**Published:** 2021-06-07

**Authors:** Bodo C. Melnik, Wolfgang Stremmel, Ralf Weiskirchen, Swen Malte John, Gerd Schmitz

**Affiliations:** 1 Department of Dermatology, Environmental Medicine and Health Theory, University of Osnabrück, D-49076 Osnabrück, Germany; sjohn@uos.de; 2 Private Praxis for Internal Medicine, Beethovenstraße 2, D-76530 Baden-Baden, Germany; wolfgangstremmel@aol.com; 3 Institute of Molecular Pathobiochemistry, Experimental Gene Therapy and Clinical Chemistry (IFMPEGKC), RWTH University Hospital Aachen, D-52074 Aachen, Germany; rweiskirchen@ukaachen.de; 4 Institute for Interdisciplinary Dermatological Prevention and Rehabilitation (iDerm), University of Osnabrück, D-49076 Osnabrück, Germany; 5 Institute for Clinical Chemistry and Laboratory Medicine, University Hospital of Regensburg, University of Regensburg, D-93053 Regensburg, Germany; gerd.schmitz@ukr.de

**Keywords:** adipogenesis, DNA methyltransferase 1, immune tolerance, intestinal maturation, milk exosome, milk miRNAs, necrotizing enterocolitis, nuclear factor-κB, receptor-interacting protein 140, systemic milk effects

## Abstract

Multiple biologically active components of human milk support infant growth, health and development. Milk provides a wide spectrum of mammary epithelial cell-derived extracellular vesicles (MEVs) for the infant. Although the whole spectrum of MEVs appears to be of functional importance for the growing infant, the majority of recent studies report on the MEV subfraction of milk exosomes (MEX) and their miRNA cargo, which are in the focus of this review. MEX and the dominant miRNA-148a play a key role in intestinal maturation, barrier function and suppression of nuclear factor-κB (NF-κB) signaling and may thus be helpful for the prevention and treatment of necrotizing enterocolitis. MEX and their miRNAs reach the systemic circulation and may impact epigenetic programming of various organs including the liver, thymus, brain, pancreatic islets, beige, brown and white adipose tissue as well as bones. Translational evidence indicates that MEX and their miRNAs control the expression of global cellular regulators such as DNA methyltransferase 1—which is important for the up-regulation of developmental genes including insulin, insulin-like growth factor-1, α-synuclein and forkhead box P3—and receptor-interacting protein 140, which is important for the regulation of multiple nuclear receptors. MEX-derived miRNA-148a and miRNA-30b may stimulate the expression of uncoupling protein 1, the key inducer of thermogenesis converting white into beige/brown adipose tissue. MEX have to be considered as signalosomes derived from the maternal lactation genome emitted to promote growth, maturation, immunological and metabolic programming of the offspring. Deeper insights into milk’s molecular biology allow the conclusion that infants are both “breast-fed” and “breast-programmed”. In this regard, MEX miRNA-deficient artificial formula is not an adequate substitute for breastfeeding, the birthright of all mammals.

## 1. Introduction

Breastfeeding is considered to represent the ideal source of infant nutrition. During the postnatal period, the infant´s epithelial barrier of the gastrointestinal (GI) tract matures, while adaptive immunity is still developing [1]. Accumulating evidence indicates that human milk (HM) is critically involved in the regulation of intestinal maturation and immune cell education [2,3]. Multiple biologically active components of HM and various interacting signaling pathways drive developmental processes which remain largely obscure [4].

**Figure 1:**
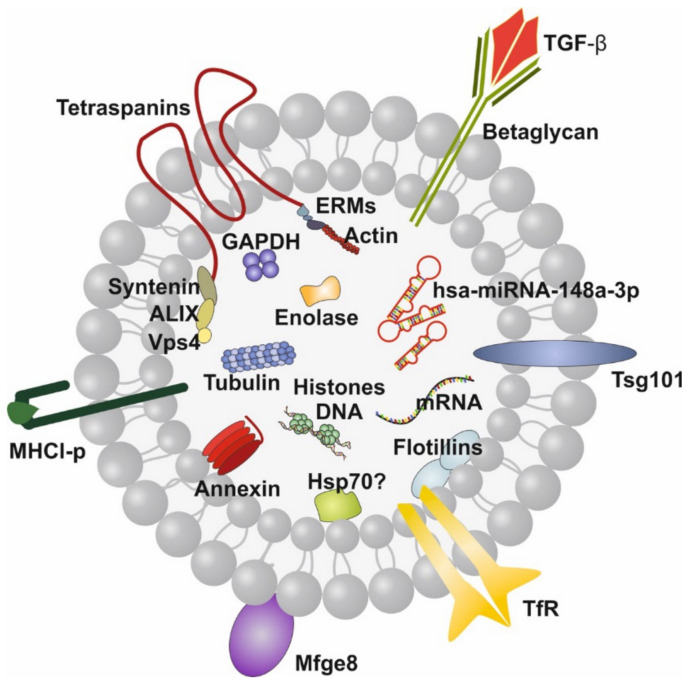
Illustration of a human milk exosome (MEX). The bilayer membrane is important for MEX resistance against the harsh conditions in the gastrointestinal tract. hsa-miRNA-148a-3p is the dominant miRNA of MEX. Note, MEX also contain transforming growth factor-β (TGF-β). Tetraspanins are CD9, CD63, CD81 and CD83.

Recently, attention has been paid to the wide spectrum of lipid bilayer-enclosed milk extracellular vesicles (MEVs), especially the subfraction of milk exosomes (MEX) that contain proteins, lipids, mRNAs, microRNAs (miRNAs), circular RNAs (circRNAs) and long non-coding RNAs (lncRNAs). Our perception that milk is not “just food” for the growing infant but represents a complex metabolic and endocrine signaling system for postnatal growth and programming via transfer of mTORC1-activating amino acids and gene-regulatory miRNAs [5,6,7] has been substantiated in recent years. HM compared to other body fluids represents the richest source of miRNAs [8]. A large spectrum of bovine MEVs isolated by differential ultracentrifugation and their miRNA composition has recently been characterized [9,10]. MEX, a most important subfamily of MEVs, are biomolecular nanostructures released from mammary gland epithelial cells (MGECs), carrying specific biomolecular information. These nanosized particles 30–150 nm in diameter precipitate in the 100,000× *g* fraction of milk [9,10]. MEX are derived from the endolysosomal pathway and are released by intraluminal budding of multivesicular bodies with the cell membrane of MGECs. They are characterized by the proteins CD9, CD63, CD81, CD82, HSP70, HSP90, Alix, TSG101, annexin and Rab GTPases, among others. MEX are found in the milk of all mammals including HM and have received increasing scientific attention in recent years [11,12]. HM transfers functionally important miRNAs that primarily originate from human MGECs, resulting in unique miRNA profiles of fractionated HM [13]. MEX survive the harsh and degrading conditions in the gut, are taken up by various cell types, cross biological barriers and reach the blood circulation and peripheral tissues [14,15,16,17,18,19]. The most abundant miRNAs of milk fat-depleted HM is miRNA-148a followed by miRNA-30a, miRNA-146b, miRNA-200a, miRNA-21, miRNA-200c, miRNA-26a, let-7f, let-7i and miRNA-146a [20,21]. miRNA-148a is also the most abundant miRNA of human MEX, accounting for almost 24% of total MEX miRNA and about 12% of miRNAs in whole HM [22] (Figure 1).

miRNA-148a is also the most abundant miRNA of triacylglycerol-rich milk fat globules (MFGs) of HM [23]. Of note, miRNA-148a promotes triacylglycerol synthesis in MGECs [24]. Remarkably, the abundantly expressed miRNAs of human MEX exhibit striking nucleotide sequence homologies with the corresponding milk miRNAs of other mammals [20,25]. It has recently been demonstrated that the top 10 highly expressed MEX-derived miRNAs are evolutionarily conserved across the milk of various mammalian species, including humans [26]. miRNA-148a, which presents the most abundant miRNA packaged into MEX [27,28], targets the mRNA of DNA methyltransferase 1 (DNMT1) [20,27,28], and thus plays a critical role in MEX-mediated epigenetic regulation [29,30,31]. miRNA-148a also belongs to the most abundant MEX-derived immune-related miRNAs of HM [32]. Notably, the immune-related miRNAs enriched in MEX are resistant to harsh environmental conditions [32]. Accumulating evidence indicates that MEX are of critical importance for intestinal, immunological, metabolic and neurological programming and cell differentiation during the postnatal period of breastfeeding [20,27,28,33,34,35,36,37,38,39,40,41]. Importantly, MEX and their miRNAs are not detectable in artificial infant formula [42].

It is the intention of this review to provide up-to-date information on the impact of MEX and MEX-derived miRNAs on intestinal maturation and their systemic effects in human and animal tissues, which are important to understand the eminent role of MEX in infant health and development.

## 2. Exosomal miRNAs and Intestinal Maturation

### 2.1. Intestinal Epithelial Cells

Cells take up exosomes by a variety of endocytic pathways, including clathrin-dependent endocytosis, and clathrin-independent pathways such as caveolin-mediated uptake, macropinocytosis, phagocytosis and lipid raft-mediated internalization [43,44,45]. Bovine MEX uptake in human and rat intestinal epithelial cells (IECs) is mediated by endocytosis and depends on cell and exosome surface glycoproteins [46]. Upon gastric/pancreatic digestion, human MEX and their overall miRNA abundance was stable and entered human intestinal crypt-like cells (HIEC) with evidence of nuclear localization [14]. As predicted earlier [29,30], Golan-Gerstl et al. [20] demonstrated that incubation of human MEX with normal colon cells (CRL1831) significantly increased cellular levels of miRNA-148a and decreased the expression of DNMT1. Furthermore, the addition of human MEX to normal fetal colon epithelial cells increased cell proliferation in an miRNA-dependent manner [27].

Insulin-like growth factor 1 (IGF-1)-mediated activation of phosphatidylinositol 3-kinase (PI3K) is negatively regulated by phosphatase and tensin homolog (PTEN). PTEN is a direct target of miRNA-148a that is down-regulated following incubation with MEX [27]. Of note, knockdown of miRNA-148a inhibits IEC proliferation associated with an increase in the expression of DNMT1 [27]. It has been demonstrated in cultured human colonic LS174T cells that exposure to bovine MEX enhances the expression of glucose-regulated protein 94 (GRP94) [47], the most abundant intraluminal endoplasmic reticulum (ER) chaperone protein. GRP94 acts as an obligatory chaperone aiding the synthesis of IGF-1, IGF-2 [48] and proinsulin [49]. Furthermore, GRP94 plays a crucial role in gut homeostasis via chaperoning crucial components of the canonical WNT pathway [50]. GRP94 interacts with Mesoderm Development (MesD), a critical chaperone for the WNT co-receptor low-density lipoprotein receptor-related protein 6 (LRP6). Without GPR94, LRP6 fails to export from the ER to the cell surface, resulting in a profound loss of canonical WNT signaling [50]. Notably, mouse models harboring intestinal knockout of GRP94 led to WNT signaling defects through loss of the WNT co-receptor LRP6, resulting in early postnatal death with loss of intestinal barrier function, decreased number of villi and significant reduction in crypts [50].

IGF-1 plays an important role in intestinal growth [51] and is a bioactive hormone of HM [52]. The IGFs and their receptors in the stomach and duodenum are expressed in all age groups and are highest in the fetus [53]. The IGF system proteins are located in the gastric glands and epithelium and in the apical portion of the villous epithelium of the duodenum. Highest IGF-1 receptor (IGF1R) expression was found in the fetal GI tract [53]. Treatment with porcine MEX promoted IPEC-J2 cell proliferation, raised mice’ villus height, crypt depth and the ratio of villus length to crypt depth of intestinal tissues. MEX also increased CDX2, PCNA and IGF1R and inhibited p53 expression [54].

IGF-1 not only promotes growth of the GI tract [51,53], but protects IECs from oxidative stress, hypoxia, thermal stress and apoptosis in the setting of intestinal injury [55,56,57,58]. Moreover, IGF-1 exerts anti-inflammatory properties [59], promotes the development and cytotoxic activity of human NK cells [60], improves intestinal barrier function [61,62] and decreases bacterial translocation [63]. IGF-1 has thus been suggested to play a promising role in the treatment or prevention of necrotizing enterocolitis (NEC) [51,64,65].

*IGF1* gene expression is induced by *IGF1* P2 promoter demethylation [66,67,68]. It has been demonstrated that DNMT1 silencing significantly increases the expression of IGF-1, whereas DNMT1 up-regulation directly results in hypermethylation of *IGF1,* thereby reducing IGF-1 expression [69]. It is thus conceivable that MEX-mediated transfer of miRNA-148a, which reduces the expression of DNMT1 in intestinal cells [20,27], may promote intestinal IGF-1 expression [29,30]. Intestinal IGF-1 expression is further promoted by MEX-mediated up-regulation of GRP94 [47], the critical chaperone for IGF-1 synthesis [48]. In accordance, MEX derived from bovine [70], porcine [54], rat [71] and yak milk [72,73] promote proliferation and survival of IECs. Notably, metabolic activity of human colorectal adenocarcinoma epithelial (Caco-2) cells after co-incubation with bovine colostrum and MEX from high immune responder cows was significantly greater than after co-incubation with MEX from low immune responder cows pointing towards immune-genetic variations of MEX bioactivity [74].

### 2.2. Intestinal Stem Cells

The intestinal epithelium is the most rapidly self-renewing tissue in mammals. Leucine-rich-repeat-containing G-protein-coupled receptor 5 (LGR5), a WNT target gene with restricted crypt expression, has been identified as marker for intestinal stem cells (ISCs) [75]. LGR5 controls fetal ISC maturation associated with acquisition of a definitive stable epithelial phenotype, as well as the capacity of ISCs to generate their own extracellular matrix [76]. Recent evidence indicates that MEX interact with ISCs. Human MEX exposure to H_2_O_2_-treated prominin-1^+^ ISCs derived from small intestines of the neonatal rat increased ISC viability compared to MEX-free controls [77]. There was a significant up-regulation of mRNA expression of LGR5, axin2, c-myc and cyclin D1 genes of the WNT/β-catenin axis in ISCs treated with human MEX as compared to controls [77]. To elucidate the mechanism by which MEX act in promoting cell growth, Hock et al. [71] investigated the gene expression levels of LGR5 in rat IEC-18 cells after incubation with rat MEX. In comparison to controls, there was a significant up-regulation of LGR5 expression in IECs treated with MEX [71]. Thus, MEX promote ISC activity, a critical mechanism for the development and maturation of the intestine during the postnatal breastfeeding period.

### 2.3. Intestinal Epithelial Barrier Function

#### 2.3.1. Tight Junctions

The intestinal epithelium establishes a selectively permeable barrier that supports nutrient absorption and waste secretion while preventing intrusion by luminal materials. Intestinal epithelia play a central role in regulating interactions between the mucosal immune system and luminal contents, which include dietary antigens, a diverse intestinal microbiome and pathogens [78]. The appropriate maturation of the intestinal permeability barrier is of critical importance for the neonate and is often immature in preterm infants, who are at increased risk for developing NEC associated with disrupted tight junctions (TJs) [79,80]. The intestinal permeability barrier is regulated by TJs, which are formed between IECs at the most apical areas of the epithelium. Formation of functional TJs is critical for the maintenance of gut permeability and intestinal barrier function [78,81]. TJs regulate the paracellular movement of molecules between the intestinal lumen and subepithelial tissues [78]. The TJ transmembrane proteins occludin, claudins and the cytoplasmic protein zonula occludens 1 (ZO-1) are considered crucial for creating the seal and thus regulate intestinal permeability [78,82,83].

Remarkably, bovine MEX derived from the 100,000× *g* ultracentrifugation fraction of commercial cow milk restored the expression of ZO-1, which was diminished by dextran sodium sulfate (DSS) in a DSS-induced murine model of colitis [84]. It has recently been demonstrated that porcine MEX attenuated deoxynivalenol (DON)-induced damage of IECs. Notably, decreased levels of ZO-1, CLDN1 and OCLN mRNA and protein in IPEC-J2 cells and the small intestinal tissues during continuous DON exposures could be significantly rescued by porcine MEX [85]. In accordance, human MEX administration 6 h prior to induction of experimental NEC, showed milder intestinal tissue injury than controls and had lower levels of pro-inflammatory cytokines and higher levels of epithelial TJ proteins ZO-1, claudin and occludin [86].

#### 2.3.2. Goblet Cells and Mucus Layer

The IECs are covered by a thick layer of mucus, which is produced by goblet cells. Mucus serves as the first line of innate host defense and provides a protective barrier that prevents microorganisms and noxious substances from reaching the surface of the epithelium [87,88]. Major components of mucus, the mucins are highly O-glycosylated molecules that have gel-like properties [87,88]. The human mucin (MUC) family is subdivided into secreted gel-forming mucins and transmembrane mucins. Secreted/gel-forming mucins such as MUC2 are responsible for the formation of the mucus layer over the epithelium, whereas the transmembrane mucins such as MUC1 are poorly understood [87,88]. Mucins are produced and stored in granules in the goblet cell cytoplasm, are transported to the cell surface and are secreted into the lumen from the apical surface of the goblet cell [89]. Mucus in the small intestine forms a diffusion barrier where anti-microbial substances keep the epithelium free from microorganisms [90]. Goblet cells are highly responsive to the signals they receive from the immune system and are also able to deliver antigens from the lumen to dendritic cells (DCs) in the lamina propria [90]. In fact, the small intestinal goblet cells can sample luminal material during mucus secretion and transfer the antigens to lamina propria DCs something that also happens in the colon if bacterial numbers are decreased. This communication with the immune system has tolerogenic effects [90]. Thus, goblet cells and their secreted mucins play a critical role in intestinal barrier function and immune homeostasis. A recent study in neonates suffering from NEC showed a significant decrease in the expression levels of MUC1, MUC2, occludin and ZO-1 as compared to healthy controls [80].

Li et al. [47] investigated the effects of bovine MEX on goblet cell expression in experimental NEC. To study the effect on mucin production, human colonic LS174T cells were cultured and exposed to bovine MEX. Compared to the control, bovine MEX promoted goblet cell activity, as demonstrated by increased mucin production and relative expression levels of goblet cell expression markers trefoil factor 3 (TFF3) and MUC2 [47]. Recently, Tong et al. [91] observed increased intestinal expression levels of MUC2 after oral administration of bovine MEVs. Reproduction of mucins was also observed after adding MEVs and MEX in the DSS-induced model of colitis associated with a restoration of lachnospiraceae and ruminococcaceae [84]. Administration of raw or Holder-pasteurized (HoP) human MEX during experimentally induced NEC in murine intestine organoids was associated with higher number of goblet cells compared to NEC without MEX exposure. Quantification of immunostaining revealed no difference in goblet cell numbers between raw and HoP human MEX. Administration of both raw and HoP human MEX during NEC increased MUC2 mRNA expression [92].

#### 2.3.3. Gut Microbiome

Among the factors influencing the mucus barrier, the microbiome plays a major role in driving mucus changes [88]. Mucus forms large pores and is penetrable to bacteria and other components, but despite this, in normal situations, the contact between bacteria and the epithelium is limited [93,94]. The continuous secretion of mucus and its flow towards the intestinal lumen donates anti-bacterial agents including lysozyme, deleted in malignant brain tumors 1 (DMBT1), immunoglobulin A (IgA), defensins, regenerating islet-derived 3γ (RegIIIγ) and phospholipase A2-IIA, which all keep bacteria away from the epithelial surface [88,95,96,97].

**Figure 2:**
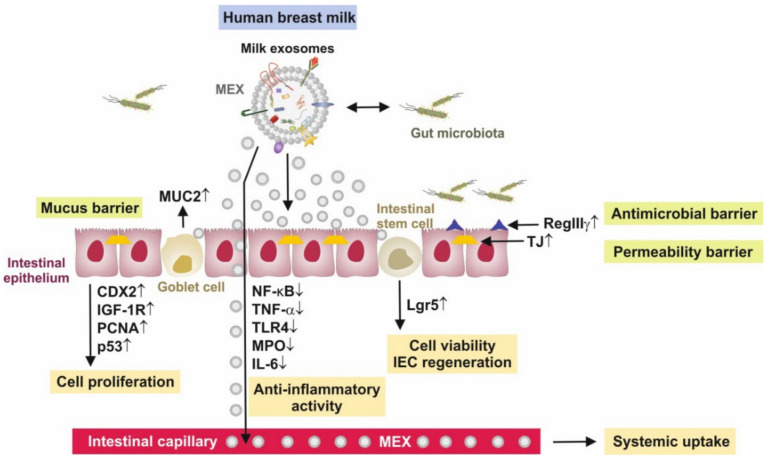
Synopsis of intestinal effects of milk exosomes (MEX). MEX increase intestinal epithelial cell proliferation, goblet cell proliferation and activity and enhance the activity and viability of intestinal stem cells with up-regulation of the stem cell marker leucine-rich-repeat-containing G-protein-coupled receptor 5 (Lgr5). MEX support the formation of the mucus barrier and increase the production of mucin 2 (MUC2), and exert anti-inflammatory activities via the suppression of nuclear factor κB signaling, tumor necrosis factor-α (TNF-α), toll-like receptor 4 (TLR4), myeloperoxidase (MPO) and interleukin 6 (IL-6). Furthermore, MEX support the anti-microbial barrier via up-regulation of the anti-bacterial lectin regenerating islet-derived 3γ (RegIIIγ) and induce the expression of tight junction (TJ) proteins zonula occludens 1, claudin and occludin. Furthermore, MEX directly interact with bacteria of the gut microbiome.

Recent evidence underlines that MEX play a significant role in the regulation of the gut microbiome (Figure 2). RegIIIγ, a secreted anti-bacterial lectin, is essential for maintaining a 50-micrometer zone that physically separates the microbiota from the small intestinal epithelial surface. The loss of host-bacterial segregation in RegIIIγ(^-/-^) mice was coupled to increased bacterial colonization of the intestinal epithelial surface and enhanced activation of intestinal adaptive immune responses by the microbiota [98]. Thus, RegIIIγ is a fundamental mechanism of innate immunity that promotes host-bacterial mutualism by regulating the spatial relationships between microbiota and host [99]. RegIIIγ expression depends on MyD88-mediated signaling downstream of toll-like receptors and the IL-1 receptor family, which is critically involved in the induction of protective host responses upon infections [100]. Functional expression of MyD88 in IECs protected mice during intestinal infection, which was associated with enhanced epithelial barrier integrity and increased expression of the RegIIIγ [100]. It has recently been demonstrated that bovine MEVs increased the expression of MyD88 and RegIIIγ [91]. Furthermore, the expression of GATA4, IgA and sIgA were increased in murine intestine after administration of bovine MEVs, which thus play a significant role in the integrity of the mucus layer and the innate intestinal defense [91]. DMBT1, a component of HM after birth, is up-regulated in HM from mothers with newborns suffering from neonatal infections [101]. It is not yet known whether MEX transport DMBT1, which has been detected in exosomes from human urine-derived stem cells promoting wound repair [102].

MEX not only interact with IECs, ISCs and goblet cells, but also shape the intestinal microbiome [103,104]. In fact, bovine MEX have been shown to alter bacterial gene expression promoting the growth of *Escherichia coli* K-12 MG1655 and *Lactobacillus plantarum* WCFS1 [103]. At the operational taxonomic units (OTU) level, four OTUs from the family of lachnospiraceae were more than two times more abundant in mice fed a bovine MEX/RNA-sufficient diet compared to mice fed a bovine MEX/RNA-deficient diet at age 7 and 47 weeks, respectively [104]. Notably, lachnospiraceae, which are butyrate-producing intestinal bacteria [105,106,107], exhibit reduced abundance in ulcerative colitis [108]. Remarkably, children with lower risk of IgE-mediated allergic diseases showed an earlier maturation of gut microbiota and an increased abundance of butyrate-producing bacteria, associated with earlier maturation of regulatory T (Treg) cells and lower IgE production [109]. The increase in highly activated Treg cells was associated with a relative abundance of *Bifidobacterium longum* followed by increased colonization with butyrate-producing bacteria [109].

### 2.4. Lamina Propria Regulatory T Cells

Treg cells are characterized by the expression of the master transcription factor forkhead box P3 (FOXP3) [110]. Intestinal Treg cells are crucial to maintain immune tolerance to dietary antigens and gut microbiota [111]. They are critical for tuning the intestinal immune response to self- and non-self-antigens in the intestine [112]. Human infants exhibit presence of mucosal FOXP3^+^ Treg cells in the small and large intestinal mucosa at birth and as early as 23 weeks of gestational age [113]. Gut-resident FOXP3^+^ CD4^+^ Treg cells are distinct from those in other organs and have gut-specific phenotypes and functions. The differentiation, migration and maintenance of intestinal Treg (iTreg) cells are controlled by specific signals from the local environment [114]. Intestinal tolerance requires gut homing and expansion of FOXP3^+^ Treg cells in the lamina propria [115]. Antigen can be acquired directly by intestinal phagocytes, or pass through enterocytes or goblet cell-associated passages prior to capture by DCs in the lamina propria. Mucin from goblet cells acts on DCs to render them more tolerogenic. A subset of regulatory DCs expressing CD103 is responsible for delivery of antigens to the draining lymph node and induction of Treg cells [116]. The equilibrium between phenotypic plasticity and stability of Treg cells is defined by the fine-tuned transcriptional and epigenetic events required to ensure stable expression of FOXP3 in Treg cells [117,118].

#### 2.4.1. Epigenetic Regulation of FOXP3 Expression

Importantly, DNA demethylation regulates stable FOXP3 expression associated with selective demethylation of an evolutionarily conserved element within the *FOXP3* locus named TSDR (Treg-specific demethylated region) [117,118,119,120,121]. In CD4^+^ T cells, the DNA methyltransferases DNMT1 and DNMT3b reside within the *FOXP3* locus and function to methylate CpG residues, thereby repressing FOXP3 expression in CD4^+^ cells, whereas complete demethylation of this site is required for stable FOXP3 expression [122]. Epigenetic regulation of *FOXP3* can be predictably controlled with DNMT inhibitors to generate functional, stable and specific Treg cells [123].

Immune-modulatory exosomal miRNA-148a-3p, along with miRNA-30b-5p, miRNA-182-5p and miRNA-200a-3p, have been designated as major immune-related miRNAs of HM [32]. MEX-derived miRNA-148a via suppressing DNMT1 might increase FOXP3 expression and intestinal FOXP3^+^ Treg cell differentiation as postulated earlier [124,125]. Exosomes play a pivotal role in important aspects of immune regulation and signaling between various cells of the immune system [126,127], especially in inflammatory bowel diseases [128,129]. In a dose-dependent manner, Admyre et al. [130] observed increased numbers of FOXP3^+^CD4^+^CD25^+^ Treg cells in peripheral blood mononuclear cells (PBMC) incubated with human MEX. In accordance, rat pups exposed to β-lactoglobulin (BLG), one of the main allergenic proteins in cow milk, in the presence of maternal rat milk developed an immune response profile similar to that of unchallenged dam-reared rats associated with a greater FOXP3 expression and increased numbers of FOXP3^+^CD4^+^ T cells [131]. In accordance, *FOXP3* TSDR demethylation was significantly lower in children with active IgE-mediated cow milk allergy (CMA) than in healthy children or those who outgrew CMA [132]. Constitutive CD4^+^CD25^+^ Treg cells alleviated clinical signs of immediate type hypersensitivity to dietary BLG by modulating the priming of BLG-specific T and B cell responses during oral sensitization [133]. Furthermore, *FOXP3* methylation was increased in peanut extract-sensitized and challenged mice pups, whereas in tolerized mice levels were significantly reduced [134]. Treg cells themselves are able to secrete miRNA-containing exosomes. Treg cells via exosome release transfer miRNAs, including let-7d, let-7b and miRNA-155, to conventional T cells. Notably, Treg cell-derived exosomal let-7d, an immune-regulatory miRNA found in human MEX [32], suppresses pathogenic T helper 1 cells [135]. Thus, *FOXP3* demethylation and consecutive Treg cell differentiation are associated with tolerance induction. MEX-mediated miRNA-148a-DNMT1 signaling may thus control the development of oral tolerance, a meaningful mechanism during weaning and the introduction of external solid foods.

Remarkably, increased Treg cell numbers are associated with raw farm milk exposure and lower atopic sensitization and asthma in childhood [136]. Of note, the protective effect of farm milk consumption on childhood asthma and atopy was lost when boiled farm milk was consumed instead of raw cow milk, pointing to a heat-labile protective factor in milk [137,138]. There is evidence that vigorous heat-treatment such as ultraheat-treatment (UHT: 135 °C, > 1 s) and boiling (100 °C) of commercial cow milk destroys MEVs and MEX and their miRNA cargo, including miRNA-148a [139,140], whereas pasteurization (72–78 °C, >15 s) of commercial milk did not affect total MEV numbers and preserved nearly 25–40% of milk´s total small RNAs, including miRNA-148a [139]. In comparison to high pressure processing of HM, HoP of HM (62.5 °C, 30 min) resulted in a significant decrease in MEX numbers [22].

Thus, early-life exposure to unpasteurized milk may protect against atopy, asthma and related conditions, independently of the place of residence and farming status, in both children and adults [141]. HM and unprocessed farm milk may enhance DNMT1-dependent stable Treg cell maturation. A recent randomized controlled trial showed that preterm neonates who received bovine colostrum had higher FOXP3 Treg cell levels compared to controls [142].

#### 2.4.2. Transforming Growth Factor β and FOXP3 Expression

Interleukin 2 (IL-2) and transforming growth factor-β1 (TGF-β1) also play a central role in Treg cell homeostasis. Naïve T cells after in vitro stimulation in the presence of TGF-β and IL-2 differentiate into iTreg cells [143,144,145]. Importantly, T cell receptor (TCR) and TGF-β signaling converge on DNMT1 to control *FOXP3* methylation and iTreg cell differentiation [146]. TCR activation causes the accumulation of DNMT1 and DNMT3b and their specific enrichment at the *FOXP3* locus, which leads to increased CpG methylation inhibiting *FOXP3* transcription. During this process, the augmentation of DNMT1 is regulated through at least two post-transcriptional mechanisms. Strong TCR signal inactivates glycogen synthase kinase 3 (GSK3) to rescue DNMT1 protein from proteasomal degradation and suppresses miRNA-148a to derepress DNMT1 mRNA translation [146]. In contrast, TGF-β signaling antagonizes DNMT1 accumulation via activation of p38 MAP kinase [146]. Interestingly, commercial cow milk contains MEX expressing immune-regulatory TGF-β [147]. In the DSS-induced murine colitis model, administration of human MEX down-regulated DNMT1 and DNMT3, whereas TGF-β was up-regulated in the colon [148]. TGF-β2 is significantly up-regulated in human MEX during weaning/early involution [149]. TGF-β in colostrum may prevent the development of atopic disease during exclusive breastfeeding and promote specific IgA production in human subjects [150]. Of note, TGF-β1 was significantly less secreted into mature milk of allergic mothers compared to non-allergic mothers [151].

miRNA-155 is another miRNA necessary for the development of Treg cells [152,153]. Notably, miRNA-155 is highly expressed in human and bovine milk [154,155]. miRNA-155 via targeting signal transducer and activator of transcription 1 (SOCS1) may activate IL-2/STAT5 signaling which promotes Treg cell development [156,157]. Both FOXP3 and TGF-β increase the expression of miRNA-155 [152,158,159], which plays a key role in the activation and differentiation of iTreg and thymic Treg (tTreg) cells [152,153].

Recent evidence indicates that the expression of uncoupling protein 3 (UCP3) is involved in the regulation of Treg cells [160]. When compared to UCP3^+/+^ mice, CD4^+^ T cells from UCP3^-/-^ mice had increased FOXP3 expression under iTreg cell conditions [160]. Notably, UCP3 is a direct target of miRNA-148a. MEX via transfer of miRNA-148a, miRNA-155 and TGF-β synergistically stimulate Treg differentiation, inducing and maintaining a tolerogenic intestinal environment that reduces the risk of intestinal inflammation and allergic sensitization [124,125].

### 2.5. Anti-Inflammatory Action of Milk Exosomes

It has been demonstrated in various experimental models of NEC that the addition of human, bovine and porcine MEX attenuated the expression of inflammatory cytokines such as interleukin 6 (IL-6), interleukin 1β (IL-1β) and tumor necrosis factor-α (TNF-α) [84,92,148,161,162], TLR4 [162,163] and nuclear factor κB (NF-κB) [162]. Previous studies showed that miRNA-146a, miRNA-155, miRNA-125b and miRNA-21, abundant immune-regulatory miRNAs of human and bovine milk and MEX [14,22,25,32,164], inhibit TLR-triggered production of inflammatory cytokines [165,166,167,168,169].

In phagocytes, changes in cytosolic Ca^2+^ regulate receptor-mediated endocytosis, phagosome-lysosome fusion and antigen processing. Calcium/calmodulin-dependent protein kinase IIα (CaMKIIα), is an important regulator of the maturation and function of DCs [170]. There is recent evidence that miRNAs are critically involved in the regulation of DC differentiation and function [171]. Importantly, miRNA-148a targets the mRNA of *CAMK2A*, the gene encoding CaMKIIα [172]. In fact, miRNA-148a-mediated inhibition of CaMKIIα inhibited the production of cytokines including IL-12, IL-6, TNF-α and IFN-β up-regulation of MHC class II expression and DC-initiated antigen-specific T cell proliferation [172]. CaMKII inhibitors blocked the antigen-induced increase in total cellular MHC class molecules as well as their trafficking to the plasma membrane, which was associated with decreased presentation of particulate and soluble MHC class II-restricted antigen [170,173]. CaMKII has been identified as an activator of IκB kinase (IKK) specifically in response to TCR stimulation [174]. CaMKII is critically involved in TCR signaling and CARD-containing MAGUK protein 1 (CARMA1)-induced NF-κB activation [175]. CaMKII-mediated phosphorylation of CARMA1 may play a key role in TCR-mediated NF-κB activation [175].

Furthermore, miRNA-148a was found to be a direct repressor of IκB kinase β (IKKβ) encoded on *IKBKB* [176]. IKKβ is a key regulator of NF-κB signaling. IKKβ via phosphorylation of IκB results in dissociation of IκB from NF-κB allowing NF-κB translocation to the nucleus, which induces the synthesis of pro-inflammatory cytokines [177]. Apparently, MEX-derived miRNA-148a, miRNA-146a, miRNA-155, miRNA-125b and miRNA-21 in a synergistic fashion negatively regulate the activation of immune cells and prevent over-activation of immune responses. MEX miRNA-148a-mediated suppression NF-κB signaling may play a key role in the regulation of immune homeostasis and intestinal inflammation [178].

**Figure 3:**
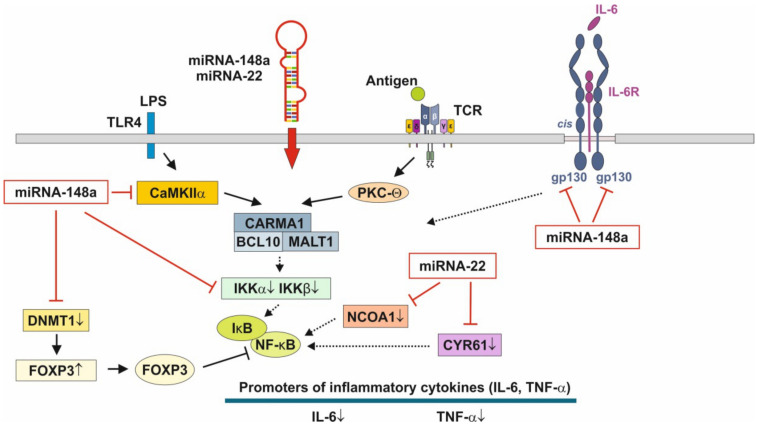
Anti-inflammatory actions of miRNA-148a and miRNA-22 on nuclear factor κB signaling. miRNA-148a via suppression of DNA methyltransferase 1 (DNMT1) enhances the expression of FOXP3, which is a negative regulator of nuclear factor κB. miRNA-148a directly targets calcium/calmodulin-dependent protein kinase IIα (CaMKIIα), which phosphorylates CARD-containing MAGUK protein 1 (CARMA1) involved in the activation of IκB kinase α (IKKα) and IκB kinase β (IKKβ), Notably, miRNA-148a directly targets IKKα and IKKβ, thereby enhancing the inhibitory effect of IκB on NF-κB. In addition, miRNA-148a targets the interleukin 6 (IL-6) signal transducer gp130. miRNA-22, which is highly expressed in preterm MEX, targets nuclear receptor co-activator 1 (NCOA1) and cystein-rich protein 61 (CYR61), which both activate NF-κB. miRNA-30b via targeting RIP140 suppresses IL-6 expression. MEX-derived miRNAs thus provide anti-inflammatory signaling.

Of note, FOXP3, which is up-regulated by miRNA-148a-mediated suppression of DNMT1 [124,125], physically associates with the Rel family transcription factors, nuclear factor of activated T cells (NFAT) and NF-κB, and blocks their ability to induce the endogenous expression of key pro-inflammatory cytokine genes [179,180]. Thus, miRNA-148a, the most abundant miRNA of HM and MEX [22], interrupts NF-κB signaling at multiple immune-regulatory checkpoints: CaMKII, IKKβ and FOXP3 (Figure 3). As the crucial inhibitory effect of glucocorticoids on NF-κB signaling relies on the induction of IκB, which traps activated NF-κB in inactive cytoplasmic complexes [181,182,183], MEX-derived miRNA-148a operates in a synergistic fashion with corticosteroids maintaining high cellular levels of IκB that attenuates pro-inflammatory NF-κB signaling.

### 2.6. Adaptive Maternal Responses of Milk Exosomes in Preterm Infants

The expression of miRNAs in the lipid and skim milk fractions of preterm HM differs significantly from term HM fractions one month after delivery. Carney et al. [184] reported nine miRNAs “altered” across both fractions and these miRNAs target a number of transcripts involved in metabolic processes. The pathway with the most significant enrichment in miRNA targets from preterm HM is glycosphingolipid biosynthesis [184], which is important for neurodevelopment, membrane function and signal transduction of lipid rafts [184]. Kahn et al. [15] demonstrated significant differences in MEX miRNA composition between the HM of mothers delivering preterm infants compared to the HM produced for term infants. The abundant miRNAs in preterm MEX are similar to those from term MEX, whereas 21 low abundance miRNAs are specifically expressed in preterm MEX compared to early term MEX [15]. Notably, miRNA-22 is highly expressed in extremely preterm MEX followed by miRNA-148a [15].

miRNA-22 is an evolutionally conserved miRNA that is highly expressed in the heart and plays an important role in cardiac remodeling [185]. Furthermore, miRNA-22 is involved in the regulation of metabolism, energy expenditure and immune functions. Important targets of miRNA-22 are PTEN, purine rich element binding protein B (PURB), caveolin 3 (CAV3), histone deacetylase 4 (HDAC4), peroxisome proliferator-activated receptor-γ co-activator 1α (PGC-1α), peroxisome proliferator-activated receptor-α (PPARα) and sirtuin 1 (SIRT1), which coordinate fatty acid metabolism, mitochondrial biogenesis and energy homeostasis [186,187]. Loss of miRNA-22 reduces fat mass gain induced by high-fat diet and enhanced energy expenditure [186,188].

miRNA-22 exerts strong anti-inflammatory activities via targeting the mRNA of cysteine-rich protein 61 (CYR61/CCN1) [189], a component of the extracellular matrix, which is produced and secreted by several cell types including endothelial cells, fibroblasts and smooth muscle cells. miRNA-22 directly targets the 3′-untranslated (3′UTR) region of the messenger RNA of *CYR61* [189] and has been implicated in leukocyte migration and inflammation (Figure 3) [190]. miRNA-22 was found to be one of the most significantly up-regulated miRNAs in LPS-stimulated RAW264.7 macrophages after treatment with simvastatin [191]. CYR61 was mainly up-regulated in intestinal mucosa after intestinal ischemia/reperfusion injury in pigs [192]. In addition, miRNA-22 attenuates myocardial ischemia-reperfusion injury via an anti-inflammatory mechanism in rats [193] and via targeting CREB binding protein (CBP) protects against myocardial ischemia-reperfusion injury through anti-apoptosis in rats [194].

It has been demonstrated in murine macrophages that CYR61 activates NF-κB-mediated transcription, and induces a pro-inflammatory genetic program characteristic of classically activated M1 macrophages that participates in Th1 responses. The effects of CYR61 include up-regulation of TNF-α, IL-1α, IL-1β, IL-6 and IL-12b [195]. miRNA-22 over- expression significantly inhibited NF-κB activity by decreasing nuclear receptor co-activator 1 (NCOA1) expression (Figure 3) [196,197]. Furthermore, miRNA-22 suppresses the function of DCs via targeting p38 mRNA [198]. p38 down-regulation reduced the synthesis of DC-derived IL-6 and the differentiation of DC-driven Th17 cells [198]. Thus, over-expressed miRNA-22 in MEX delivered to preterm infants with low birthweight appear to promote growth, weight gain, tissue maturation and attenuates inflammatory responses. This suggests that preterm milk and their MEX-derived miRNAs may have adaptive functions for growth and maturation in premature infants.

circRNAs and lncRNAs are also delivered by human, bovine and porcine MEX, that are stable to in vitro digestion [199,200,201]. circRNAs have been shown to promote ISC self-renewal [202]. Murine and human Lgr5^+^ ISCs showed high expression of the immune cell-associated circRNA circPan3 [202]. circRNAs are related to inflammatory bowel disease and intestinal barrier formation [203]. Recent studies have shown that exosomal circRNAs play critical roles in the development of neonatal tissue and organ, such as the brain [204] and the nervous system [205]. Zhou et al. [199] identified 6756 circRNAs both in preterm human colostrum and term colostrum, of which 66 were up-regulated and 42 were down-regulated in preterm colostrum. Pathway analysis showed that the vascular epithelial growth factor (VEGF) signaling pathway was involved. In particular, MEX found in preterm colostrum and term colostrum promoted VEGF protein expression and induced the proliferation and migration of small IECs [199].

Taken together, the physiological adaptations of colostrum and MEX RNAs in the milk of mothers, who delivered preterm infants, may accelerate intestinal maturation, barrier function and innate immunity, critical factors for the prevention of NEC.

## 3. Necrotizing Enterocolitis

### 3.1. Pathogenesis

Necrotizing enterocolitis (NEC), which mostly affects premature neonates, is a life-threatening inflammatory intestinal disease that can result in sepsis, multiorgan failure, short gut syndrome, which requires long-term intravenous nutrition, subsequent liver damage and death [206,207,208]. Prematurity, formula feeding [209], systemic stress, sepsis, hypoxia [209], aberrant microbiome (dysbiosis) [210,211], deviated innate immunity with exaggerated NF-κB signaling [210,212,213], intestinal ischemia and necrosis and gut barrier disruption [214] all lead to fulminant organ failure. Prematurity and formula feeding have been emphasized as major risk factors for NEC [209]. Formula feeding and immature gut microcirculation promote intestinal hypoxia promoting NEC. One of the most important pathways that mediates the balance between injury and repair in the premature intestine, and that plays a key role in NEC pathogenesis, is TLR4, which recognizes lipopolysaccharide (LPS) on Gram-negative bacteria [215]. TLR4 promotes NEC by inducing inflammation, inhibiting enterocyte proliferation and reducing intestinal microcirculation [216,217]. Indeed, formula feeding and systemic hypoxia synergistically induced intestinal hypoxia in experimental NEC [218]. TLR4-dependent Th17 polarization was required for NEC development, as inhibition of STAT3 or IL-17 receptor signaling attenuated NEC in mice, while IL-17 release impaired enterocyte TJs, increased enterocyte apoptosis and reduced enterocyte proliferation, leading to NEC [216]. Recently, a higher proportion of CCR9^+^ CD4^+^ T cells occurred in peripheral blood of both patients and mice with NEC as compared to controls [219]. Elevated CCR9^+^ CD4^+^ T cells were primarily CCR9^+^ IL-17-producing Treg cells, possessing features of conventional Treg cells, but their suppressive activity was seriously impaired and negatively correlated with the severity of intestinal tissue injury. IL-6 promoted polarization of CCR9^+^ Treg cells to CCR9^+^ IL-17-producing Treg cells, and blocking IL-6 signaling inhibited this conversion in vitro and ameliorated experimental NEC in vivo [219]. With or without LPS stimulation, monocytes from NEC infants presented elevated TNF-α and IL-6 expression, together with reduced expression of TGF-β [220]. When incubated with autologous CD4^+^ T cells, monocytes from NEC infants preferentially promoted the differentiation of RORγt-expressing Th17 cells, but not FOXP3-expressing Treg cells [220]. However, using exogenous TGF-β and IL-10, the development of FOXP3 expression could be significantly elevated [220]. Notably, a paucity of IL-10 in HM was found in mothers, whose infants developed NEC [221]. TGF-β, a strong immune suppressor and a constituent in HM and MEX [147,222], has been shown to be protective against NEC [223]. TGF-β1 suppressed NF-κB activation, maintained levels of the NF-κB inhibitor IκBα in the intestinal epithelium and systemically decreased serum levels of IL-6 and IFN-γ [223]. HM feedings, both from maternal and human donor milk, have been associated with reductions in NEC in preterm infants [224]. Among the various NEC-protective factors delivered by HM, MEX appear to play a dominant role.

### 3.2. Milk Exosomes in Experimental Necrotizing Enterocolitis

While no animal model perfectly mimics human NEC, each has greatly improved our understanding of this serious disease [225]. Nutritional modulation of the gut microbiota and immune system in preterm neonates susceptible to NEC is important for the prevention and treatment of NEC [224,226,227]. In fact, HM is the highly recommended feeding strategy to prevent NEC [227,228]. There is recent interest in the role exosomes for intestinal mucosal immunity and inflammatory bowel diseases [229]. Particularly, MEX have emerged as key players in the pathogenesis, prognosis, prevention and treatment of NEC [230]. Human, bovine and porcine MEX have been administered in various intestinal models (IECs, rodent intestine, intestinal organoids, genetically induced colitis) and prior, during or after several types of cell injury such as oxidative stress (hydrogen peroxide), hypoxia, hyperosmolar formula, mucosa cell toxins (dioxynivalenol, dextran sulfate sodium) and inflammatory agents such as lipopolysaccharides (LPS). Table 1 presents an overview of the biological outcomes of MEX administration in various colitis and NEC models.

MEX have been demonstrated to protect IECs from oxidative stress [77,231] and hypoxia [47,72,232,233], stimulate ISC activity [77], improve the proliferation and migration of IECs [72,148,162], improve intestinal barrier function and mucin production [85,92,234], reduce intestinal inflammation [84,92,162,163,235] and lower the incidence and severity of experimental NEC [84,85,92,148,163,235]. Importantly, MEX-derived miRNA-148a and miRNA-22, two major miRNAs of colostrum and mature HM, attenuate the inflammatory cascade at critical checkpoints.

### 3.3. Anti-Inflammatory Action of miRNA-148a, miRNA-22 and miRNA-30b

miRNA-148a attenuates NF-κB signaling at various regulatory checkpoints (Figure 3). Decreased expression of miRNA-148a has been demonstrated in LPS-stimulated endometrial epithelial cells, where TLR4 has been identified as a direct target of miRNA-148a [236]. In this model, over-expression of miRNA-148a using agomiR markedly reduced the production of pro-inflammatory cytokines, such as IL-1β and TNF-α, and suppressed NF-κB p65 activation by targeting the TLR4-mediated pathway [236]. Remarkably, Zhu et al. [237] demonstrated that miRNA-148a inhibits colitis and colitis-associated tumorigenesis in mice. miRNA-148a is down-regulated in human inflammatory bowel disease (IBD) and colorectal cancer (CRC) patient tissues [237]. Of note, miRNA-148a-3p negatively regulates CRC tumor cell PD-L1 expression. Decreased levels of miRNA-148a-3p have been associated with an immunosuppressive tumor microenvironment [238]. Drastically reduced miRNA-148a-3p/5p expression was observed in the colons after DSS treatment of mice [237]. miRNA-148a directly targets several well-accepted upstream regulators of NF-κB and STAT3 signaling, including GP130, IKKα, IKKβ, IL1R1 and TNFR2, which leads to decreased NF-κB and STAT3 activation in macrophages and colon tissues [237]. GP130, also known as IL-6 signal transducer [239], is conserved in the IL-6 family of cytokines [240] and plays a key role in pro-inflammatory IL-6/GP130-STAT3 signaling (Figure 3) [241,242].

The nuclear receptor-interacting protein 140 (RIP140) promotes the activity of NF-κB and up-regulates the expression of pro-inflammatory genes such as TNF-α and IL-6 [243]. The function of RIP140 as a co-activator for cytokine gene promoter activity relies on direct protein–protein interactions with the NF-κB subunit RelA and histone acetylase cAMP-responsive element binding protein (CREB)-binding protein (CBP) [243]. Of importance, miRNA-30b-5p, a key immune-regulatory miRNA of human MEX [32], targets the 3′UTR region of the mRNA of RIP140 (*NRIP1*) [244], thus attenuates pro-inflammatory action of NF-κB.

These findings reveal that miRNA-148a exerts strong anti-inflammatory activities and is an indirect tumor suppressor that modulates colitis and colitis-associated tumorigenesis by suppressing the expression of signaling by NF-κB and STAT3 and their pro-inflammatory consequences. In contrast to prostate cancer [245,246,247,248], commercial milk consumption has been epidemiologically associated with a reduced risk of colon cancer [249,250,251]. Notably, up-regulated expression of miRNA-148a has been detected in prostate cancer tissue [252], whereas reduced expression of miRNA-148a is a hallmark of colon cancer [253,254]. In contrast to the anti-inflammatory effects of MEX, recently identified bovine meat and milk factors (BMMFs), which are small episomal DNA molecules isolated from milk also from colon cancer tissue, were related to local chronic intestinal inflammation promoting CRC [255,256].

Reif et al. [27] demonstrated that human MEX entered CCD841 normal colon epithelial cells (CCD841) and colonic cancer cells (LS123) and increased intracellular miRNA-148a levels. In contrast to colonic tumor cells, MEX stimulated proliferation of CCD841 normal colon epithelial cells under starvation conditions [27].

MEX-mediated attenuation of the severity of DSS-induced colitis associated with a reduced the histopathological scoring grade and reduced expression of IL-6 and TNF-α [148] may be well explained by the anti-inflammatory action of MEX-derived miRNA-148a.

### 3.4. Hormonal Regulation of MiRNA-148a Expression

There is recent interest in the use of melatonin in oxidative stress-related neonatal diseases including NEC [257,258,259,260]. Highest melatonin concentrations were detected in colostrum, followed by transitional and mature HM [261]. Notably, melatonin ameliorates NEC in a neonatal rat model decreasing TNF-α and IL-1β [262]. Recent evidence in an NEC mouse model indicates that melatonin treatment ameliorates inflammation and improves intestinal Th17/Treg cell balance [263]. It has recently been demonstrated in MDA-MB-231 cells that melatonin increased the expression of miRNA-148a [264]. Thus, maternal melatonin may modify the expression of anti-inflammatory miRNA-148a in MEX, a potentially supportive effect of melatonin in HM that may induce IEC miRNA-148a expression.

Lactogenic hormones also alter cellular and extracellular miRNA expression in MGECs [265]. Dexamethasone, insulin and prolactin induced lactogenic differentiation of bovine MGECs associated with increased expression of miRNA-148a [265].

Oxytocin is another important hormone of perinatal physiology and component of HM [266]. Recent evidence indicates that colostrum oxytocin modulates cellular stress response, inflammation and autophagy markers in newborn rat gut villi [267]. Notably, colostrum increased inactive p-eIF2a, p-PKR and IκB and reduced p-IκB, BiP and LC3A. LPS increased and oxytocin decreased p-IκB underlining the anti-inflammatory activity of oxytocin on the postnatal gut [267]. In accordance, administration of oxytocin decreased, while the oxytocin receptor antagonist atosiban exacerbated intestinal inflammation in murine experimental model of NEC [268]. It is thus tempting to speculate that oxytocin in concert with melatonin and prolactin may augment MEX miRNA-148a expression.

### 3.5. MEX-Mediated Up-Regulation of TNF-α-Induced Protein 3

Increasing densities of *Clostridium perfringens* have been associated with NEC in preterm infants [269,270]. Total bacterial and *C. perfringens* densities were higher in NEC versus healthy pigs and correlated positively with NEC severity. In IPEC-J2 cells, expression levels of inflammation-related genes (*CCL5*, *NFKBIA*, *IL8*, *IL1RN*) and TNF-α-induced protein 3 (*TNFAIP3*) increased, while the expression of the sodium/glucose cotransporter (*SLC5A1*) decreased, with increasing density of *C. perfringens* [271]. It has been demonstrated that *C. perfringens* type C activates TLR4/MyD88/NF-κB signaling in piglet small intestines [272]. TNFAIP3, also known as A20, is a deubiquitinase which operates as a key negative regulator of NF-κB signaling that is essential for maintaining immune homeostasis and down-regulating inflammation [273,274]. Mice lacking A20 specifically in DCs (pre-colitic A20fl/fl Cd11c-Cre mice) spontaneously developed lymphocyte-dependent colitis and exhibited an increased susceptibility to DSS-induced colitis. Taken together, these results indicate that DCs require A20 to maintain intestinal immune homeostasis and to restrict epithelial damage-triggered colitis [275]. Importantly, Benmoussa et al. [84] observed a significant increase in A20 expression and decrease in miRNA-125b in DSS-induced murine colitis after administration of bovine MEX supporting the anti-inflammatory activity of MEX in this model of NEC. Notably, increased miRNA-125b expression has been reported in the DSS-induced colitis model, which is suppressed by milk MEVs [84].

### 3.6. Milk Exosome Lipidomics and NEC Prevention

Lipid encapsulation of MEX cargo protects miRNAs and other sensitive components against the harsh conditions in the GI tract [34]. Exosomes are often enriched in cholesterol, sphingomyelin, glycosphingolipids and phosphatidylserine compared to their donor cells. Lipids not only have a structural role in exosomal membranes but also are essential players in exosome formation and release to the extracellular environment and cell signaling [276]. A total of 395 lipids are identified in term and preterm human MEX [277]. Notably, phosphatidylethanolamine (18:1/18:1), phosphatidylcholine (18:0/18:2), phosphatidylcholine (18:1/16:0), phosphatidylserine (18:0/18:1) and phosphatidylserine (18:0/22:6) were enriched in term and preterm human MEX [277]. Pathway analysis indicated that MEX lipids were associated with the ERK/MAPK pathway. LPS treatment increased the expression level of p-ERK, which was reduced after treatment with term and preterm human MEX, suggesting that human MEX lipids may ameliorate NEC phenotypes via the ERK/MAPK signaling [277].

It has recently been demonstrated that HM-specific lipid mediators referred to as 1-O-alkyl-sn-glycerol ether lipids maintain beige adipose tissue (BET) in infants and prevent the transdifferentiation of BET into lipid-storing white adipose tissue (WAT) [278]. These aklylglycerols are deficient in cow milk-based infant formula [278].

Various ether lipids have been detected in exosomes [276]. Notably, it has been shown in exosomes derived from PC-3 cells, that the ether lipid precursor hexadecylglycerol stimulates exosome release and changes the protein composition of exosomes [279]. It is thus conceivable that alkylglycerols derived from MGECs may enhance the release of alkylglycerol-loaded MEX that may modify macrophage differentiation [280,281] involved in the transition of BET into WAT [278,282]. MEX may thus function like exosomes from adipose-derived stem cells (ADSC) that attenuate adipose inflammation and obesity through polarizing M2 macrophages and beiging in WAT [282]. MEX-like ADSC-derived exosomes may promote BET, which is important for the maintenance of the body’s core temperature of the newborn infant by heat generation due to an uncoupling mitochondrial terminal electron transport chain from ATP generation [283,284].

### 3.7. Improvement of Malnutrition-Induced Intestinal Barrier Dysfunction

Recent evidence in mice fed low protein (1%) diet showed that malnutrition-induced intestinal villus atrophy and barrier dysfunction could be rescued by oral gavage of bovine MEVs (132 nm; CD9-, CD63-, CD81-positive) [285]. Despite continued low protein diet feeding, MEV/MEX treatment improved intestinal permeability, intestinal architecture and cellular proliferation [285]. These data suggest that MEV administration may be of therapeutic value for the clinical management of malnourished children who are at high risk for morbidity and mortality.

## 4. Systemic Bioavailability of Milk Exosomes for Epigenetic Regulation

Despite an ongoing controversial scientific debate [7,286], accumulating evidence supports the systemic uptake and tissue distribution of MEX and their miRNAs in various animal models [18,19,161,287] and healthy human volunteers [288,289], providing the rationale for the therapeutic use of MEX [12,17,290,291,292,293,294,295,296]. The suitability of exosomes as delivery vehicles for extracellular RNAs was tested by evaluating the absorption of miRNA-148a-3p in hepatic and intestinal cell lines [296]. The appearance of bovine MEX and MEX-derived miRNAs in various murine tissues after oral administration of bovine MEX [18,19], the detection of specific bovine MEX miRNAs in the serum of piglets fed on cow milk underline the systemic bioavailability of MEX [287]. Furthermore, the dose-related increase in selected miRNAs in the plasma and blood monocytes of healthy human individuals after consumption of commercial pasteurized cow milk underline the systemic availability of MEX [294,295]. Accumulated evidence confirms the systemic bioavailability of MEX and their gene-regulatory actions [7,29,30,36,288,289,297]. The period of development that extends from pre-conception to early infancy is the period of life during which epigenetic DNA imprinting activity is the most active [298,299]. The early postnatal period of humans is a critical developmental epigenetic window [300,301]. Physiologically, this critical time period is controlled by signals derived from the human but not from the bovine lactation genome or artificial feeding systems [302,303]. Accumulating evidence identifies MEX as most critical signalosomes of HM that modify postnatal epigenetic regulation [29,30,31]. MEX-derived miRNA-148a suppresses DNA methylation via targeting DNMT1, which is responsible for maintenance DNA methylation, ensures the fidelity of replication of inherited epigenetic patterns. Notably, DNMT1 has a very distinguishable preference of methylating CpGs on hemimethylated DNA [304,305].

### 4.1. Milk Exosomes, Thymic T-Cell Maturation and Atopy Prevention

Evidence derived from a recent systematic review suggests that feeding HM for short durations or not at all is associated with higher childhood asthma risk [306], whereas breastfeeding offers advantages for the prevention of allergic diseases [307,308]. Maternal atopy is highly associated with food sensitivity among children who were born via Cesarean section and were non-exclusively breastfed, whereas no association among children who were vaginally delivered and exclusively breastfed was found [309].

Epigenetic changes and the potential of maternal and postnatal nutrition on the development of allergic disease are in the focus of recent research. Allergic sensitization of mothers modifies their MEX composition. Significantly lower levels of MUC1 were detected on CD63-enriched MEX from sensitized mothers compared with nonsensitized [310]. Notably, mothers whose children developed sensitization had an increased amount of HLA-ABC on their MEX enriched for CD63 [310]. Although several miRNAs (miRNA-452-5p, let-7d-3p, miRNA-146b-5p, miRNA-21-5p, miRNA-22-3p, miRNA-375, miRNA-16-5p, miRNA-511-5p, miRNA-26b-5p, let-7f-5p, miRNA-30e-5p, miRNA-374a-5p, miRNA-335-5p) were differentially expressed between offspring atopic dermatitis (AD) vs. non-AD at 2 years of age, none had an acceptable false discovery rate and their biological significance on the development of AD was not immediately apparent from functional analysis [311].

Kalliomäki et al. [150] determined the concentrations of TGF-β1 and TGF-β2 in maternal colostrum and mature milk and hypothesized that TGF-β in colostrum may prevent the development of atopic disease during exclusive breastfeeding and promote specific IgA production in human subjects. Rigotti et al. [151] reported that TGF-β1 was significantly less secreted in mature milk of allergic mothers. After 6 months, 46% infants from allergic mothers, but none from controls, presented AD [151]. Although not yet determined in human MEX, TGF-β is a constituent of bovine MEX [147].

Of note, exosomes released by mast cells harbor both active and latent TGF-β1 on their surfaces [312]. Remarkably, TGF-β1 associated with exosomes has higher signaling stability compared with free TGF-β1 and more effectively activates TGF-β1 signaling by phosphorylation of SMAD2 [312]. SMAD2/3 are required for the development of tTreg and iTreg cells [313,314,315]. SMAD2/3 and Treg-specific DNA demethylation has been shown to be important for Treg cell stability [117,118,119,120,121,122,123,125]. TGF-β is another pivotal activator of Treg cell differentiation. In an ovalbumin peptide TCR transgenic adoptive transfer model, TGF-β-converted transgenic CD4^+^CD25^+^ suppressor cells proliferated in response to immunization and inhibited antigen-specific naive CD4^+^ T cell expansion in vivo. In a murine asthma model, co-administration of these TGF-β-induced suppressor T cells prevented house dust mite-induced allergic pathogenesis in lungs [313]. In accordance, immunosuppressive Treg cells have been induced by intranasal immunization with the live-attenuated pneumococcal vaccine SPY1 via activation of TGF-β1/SMAD2/3 signaling [316]. Thus, deficient exosomal TGF-β transfer via HM of atopic mothers as well as missing exosomal TGF-β and miRNA-148a signals in artificial formula may impair Treg cell maturation increasing the risk of atopy in infants. Among the multitude of immune-regulatory bioactive compounds in HM, MEX play a key role. Exosomes are natural ancient nanoparticles of life that control critical events in immune regulation [126,317,318].

In fact, MEX belong to the complex signaling system of HM that contributes to the development of the infant’s immunity [319]. It has been demonstrated that bovine MEX cross epithelial boarders and reach various murine tissues after oral application [18,19] and may thus reach the thymus supporting MEX miRNA-mediated differentiation of tTreg cells [124,125]. Increasing evidence underlines the role of exosomes as important routes of communication within the thymus [320,321] and the induction of Treg cells [322]. It is conceivable that MEX-derived miRNA-148a via targeting DNMT1 as well as MEX-mediated transfer of TGF-β may augment stable thymic FOXP3 expression and thus tTreg cell development [130,146]. The atopy-preventive effect of raw cow milk consumption with abundant transfer of bioavailable bovine MEX [124,125] is abolished by boiling farm milk [137,141]. Indeed, boiling and UHT treatment of cow milk dramatically reduces MEX numbers and diminishes their miRNA recovery [139,140]. Notably, the TGF-β1 concentration in raw unpasteurized cow milk decreased to 50% by boiling as well [323].

### 4.2. Milk Exosomes and Hepatic Metabolism

The liver is the central organ for glucose and lipid metabolism and is a key frontline immune tissue [324,325]. Substantial accumulation of orally administered bovine MEX in murine liver have been demonstrated [18]. The neonatal crystallizable fragment receptor (FcRn) is responsible for maintaining the long half-life and high levels of the two most abundant circulating proteins, albumin and IgG [326]. Betker et al. [293] proposed that MEX can be taken up as intact particles via transcytosis involving FcRn. In fact, co-administration of bovine IgG with bovine MEX reduced intestinal absorption of fluorescent-labeled bovine MEX [293]. FcRn is significantly expressed in hepatocytes, Kupffer cells, sinusoidal epithelial cells in human liver, apical enterocytes, goblet cells and enterocytes of crypts in the small and large intestine of humans [327]. Organs with the highest FcRn expression are spleen, lymph node, liver and lung [328]. It has recently been shown that exosome-mediated intercellular communication between hepatitis C virus-infected hepatocytes and hepatic stellate cells may be critically involved in pathogenesis of liver fibrosis [329,330].

Over-expression of miRNA-148a in postnatal rat liver reduced the expression of LDL receptor (LDLR), impairing liver cholesterol reverse transport [331], a meaningful mechanism for peripheral cholesterol supply of growing tissues during the breastfeeding period. MEX-mediated transfer of miRNA-148a, which targets hepatic LDLR, may thus direct LDL to peripheral cells required for tissue growth. In a comparable fashion, MEX-derived miRNA-29b via targeting dihydrolipoamide branched chain transacylase (DBT) [332], the core component of branched chain α-ketoacid dehydrogenase, may attenuate hepatic catabolism and oxidation of branched-chain amino acids (BCAAs), directing the flux of BCAAs to BCAA-mTORC1-mediated hepatic protein and albumin synthesis [333,334,335].

### 4.3. Milk Exosomes and Neurodevelopment

Breastfeeding is associated with increased intelligence [4,336,337]. Among 285 participants, each month of exclusive feeding at the breast only was associated with a decreased risk of clinically meaningful executive function (working memory) deficit [338]. Prolonged and exclusive breastfeeding improves children’s cognitive development [339,340]. As demonstrated by Manca et al. [18], MEX accumulate in the brain of mice after oral administration of bovine MEX. In fact, recent evidence indicates that exosomes are able to cross the blood-brain-barrier [341,342,343,344]. The brain undergoes maturation in the early postnatal period. Particularly, the hypothalamic-pituitary axis, an essential regulator of food intake and energy homeostasis, is relatively immature at birth in both rats and mice [345]. During the first 2 weeks of postnatal life, hypothalamic neurons send axonal projections to their target sites and form functional synapses [345]. In early postnatal life, developmental processes are critical for establishing proper neuronal connectivity in the brain requiring the synaptic machinery.

One protein thought to be important in synaptic plasticity is α-synuclein (α-syn) [346]. Postnatal expression of α-syn is developmentally regulated suggesting that α-syn may play a pivotal role in establishing the function of basal ganglia [346]. In the rat, a high level of α-syn expression within cell bodies of the substantia nigra pars compacta is observed in the 1st week of postnatal life, which decreases both in intensity and number of immunoreactive cells between postnatal days 7 and 14 [346]. Soluble α-syn is an abundant neuronal protein that localizes predominantly to presynaptic terminals [347,348,349,350]. Monomeric α-syn promotes membrane curvature and assembly of the soluble N-ethyl-maleimide-sensitive factor attachment protein receptor (SNARE) complex, a mediator for vesicle fusion with target membranes [351,352,353].

Of note, the SNARE protein is also the molecular basis of exocytotic activity for insulin secretion [354]. In addition, α-syn contributes to synaptic trafficking, vesicle budding and vesicle recycling, while in the case of dopaminergic neurons, α-syn mediates dopamine synthesis, storage and release [355,356,357]. Furthermore, SNAREs have been proposed to facilitate the fusion of multivesicular bodies with the plasma membrane promoting exosome release [358].

Accumulating evidence underlines that hypomethylation of the *SNCA* promoter increases α-syn expression, which is controlled by DNMT1 [359,360,361,362,363,364,365,366,367,368]. α-syn itself sequesters DNMT1 from the nucleus promoting hypomethylation of *SNCA* further augmenting α-syn expression in a vicious cycle [360]. Remarkably, the depletion of dietary bovine MEX impairs sensorimotor gating and spatial learning in C57BL/6 mice fed an AIN-93G-based, bovine-MEX-deficient diet for up to 20 weeks [369].

### 4.4. Milk Exosomes and Potential Impact on Pancreatic β-Cell Proliferation

There is recent interest in the role of perinatal nutritional programming of epigenetic processes controlling energy metabolism and body composition maintenance [298,299,300,301,370]. Breastfeeding is recommended for the prevention of overweight and type 2 diabetes mellitus (T2DM) [4]. The insulin-secreting pancreatic β-cells play a central role in glucose homeostasis and metabolic regulation. During the breastfeeding period, pancreatic β-cells proliferate and extend islet β-cell mass to provide sufficient capacities for insulin secretion required for the changing demands after weaning and the introduction of solid foods [371,372,373]. Most β-cell neogenesis in humans was observed preterm with a burst of β-cell proliferation, peaking within the first 2 years of life [373]. The β-cell to α-cell ratio doubled neonatally, reflecting increased growth of β-cells [373]. It is known that the β-cells’ proliferative capacity declines postnatally, but the extrinsic cues and intracellular signals that cause this decline remain unknown [374].

Accumulating evidence supports the view that β-cells are involved in extensive exosome signaling maintaining a metabolic organ cross-talk [375,376,377,378,379,380]. It has been predicted that MEX derived from HM and pasteurized commercial cow milk may interact with pancreatic β-cells and promote β-cells proliferation, a physiological mechanism for postnatal β-cell mass expansion, but a damaging constellation when MEX-driven β-cell signaling persists [381,382]. IGF-1/mTORC1 signaling is a key driver of β-cells proliferation [383,384,385]. mTORC1 specifically regulates both proliferation and identity maintenance of neonatal β-cells [385,386]. Milk consumption enhances hepatic IGF-1 secretion and stimulates mTORC1 signaling including β-cell mTORC1 activation [6,381,382]. *IGF1* is a DNMT1-regulated developmental gene, whose expression is epigenetically enhanced by *IGF1* P2 promoter demethylation [66,67,68,69]. After weaning, Jafaar et al. [387] observed a switch from increased β-cell mTORC1 activation toward enhanced 5′-adenosine monophosphate-activated protein kinase (AMPK) signaling. The acquired AMPK-dependent adult β-cell signature was associated with an increased capacity for glucose-stimulated insulin secretion (GSIS), enhanced β-cell mitochondrial biogenesis, a shift to oxidative metabolism and functional β-cell maturation, whereas in T2DM a remarkable reversion of the normal AMPK-dependent adult β-cell signature to the more neonatal one with increased mTORC1 activation was observed [387]. It has recently been hypothesized that the shift towards higher β-cell AMPK activity after weaning might be associated with the termination MEX transfer to the infant [382]. During breastfeeding MEX-derived miRNA-148a may suppress β-cell AMPK activity via targeting the catalytic subunit α 1 of AMPK (*PRKAA1*) as well as the AMPK regulatory subunit γ 2 (*PRKAG2*) [382,388]. Further important target genes of miRNA-148a are *MAFB* (V-MAF musculoaponeurotic fibrosarcoma oncogene family, protein B), which is involved in β-cell differentiation, *ESRRG* (estrogen-related receptor-γ) and *PPARGC1A* (peroxisome proliferator-activated receptor-γ, co-activator 1α), which regulate mitochondrial function and oxidative metabolism [382]. Furthermore, loss of DNMT1, a key target of miRNA-148a, results in the conversion of pancreatic islet α-cells into β-cells [389].

Taken together, translational evidence links MEX to epigenetic regulation of β-cell proliferation and β-cell mass expansion, whereas the decline of MEX miRNA-148a signaling during the weaning period may explain the switch towards functional β-cell maturation [382]. Notably, increased plasma levels of miRNA-148a have been found in patients with T2DM exhibiting correlations with pathological oral glucose tolerance test, glycated hemoglobin (HbA1c), insulinemia and increased homeostasis model assessment for insulin resistance (HOMA-IR) [390].

Recently, aberrant levels of miRNAs have been detected in MEX of mothers with type 1 diabetes mellitus (T1DM) [391]. Nine MEX miRNAs were found differentially expressed in mothers with T1DM compared to healthy mothers. The highly up-regulated miRNAs, hsa-miR-4497 and hsa-miR-3178, increased LPS-induced expression and secretion of TNF-α in human monocytes. The up-regulated miRNA target genes were significantly enriched for longevity-regulating pathways and FOXO signaling [391].

RIP140, the key regulator of metabolic balance, plays also an important role in β-cell homeostasis and insulin secretion [392]. Over-expression of RIP140 promoted apoptosis but inhibited cell viability in MIN6 cells, and basal insulin secretion and GSIS levels were altered following treatment with glucose and palmitic acid. In addition, oxidative stress was elevated, phosphorylated extracellular signal-regulated kinases 1/2 and uncoupling protein 2 (UCP2) messenger RNA (mRNA) abundance were increased, B-cell lymphoma-2 protein levels were decreased, and PPARγ co-activator 1α, phosphoenolpyruvate carboxykinase and pancreatic and duodenal homeobox-1 mRNA levels were down-regulated. Furthermore, glucolipotoxicity-induced damage was reversed when RIP140 expression was down-regulated by small interfering RNA (siRNA) [392]. Thus, MEX miRNA-30b via suppressing RIP140 may enhance β-cell function and insulin secretion, which is important for mTORC1-dependent postnatal growth.

### 4.5. Milk Exosomes and Their Potential Impact on Beige/Brown Adipogenesis

Breastfeeding is inversely associated with a risk of early obesity in children aged 2 to 6 years. Moreover, there is a dose-response effect between the duration of breastfeeding and reduced risk of early childhood obesity [393,394,395,396]. Adipose tissue undergoes profound compositional changes in early life, of which an increased understanding could offer potential interventions to retain brown adipose tissue (BAT) in later life [397]. Like HM-derived-alkylglycerols [278], MEX may also function as signalosomes modifying the homeostasis of BET, BAT and WAT. From a teleological point of view, MEX should promote both BET/BAT and WAT to maintain adequate thermogenesis and storage of energy reservoirs.

Dominant miRNAs of HM, such as miRNA-30b, miRNA-155 and miRNA-148a, are related to adipose tissue development. Recently, Villatoro et al. [398] demonstrated that canine colostrum exosomes (CCE) modified the proliferation and secretory profiles in canine mesenchymal stem cells derived from bone marrow (cBM-MSCs) and adipose tissue (cAd-MSCs). An increase in cAd-MSCs proliferation for 12 days in the presence of CCE, whereas this effect was not observed in cBM-MSCs [397]. Kupsco et al. [40] evaluated MEX miRNA expression in relation to maternal BMI (in kg/m^2^). Of 419 miRNAs evaluated, 374 were negatively associated with BMI, whereas miRNA-4769-5p was weakly, but significantly, positively associated with BMI. The top four miRNAs most significantly negatively associated with BMI were miRNA-128-3p, miR-130a-3p, miRNA-574-3p and miRNA-6881-5p [40]. Recently, Shah et al. [399] investigated the impact of maternal overweight/obesity on selected HM MEX-derived miRNAs involved in adipogenesis and glucose metabolism to elucidate their relationship with measures of infant body composition in the first 6 months of life. Remarkably, the abundance of miRNA-148a and miRNA-30b in the overweight/obesity group was lower by 30% and 42%, respectively, compared with the control group at 1 month. miRNA-148a was negatively associated with infant weight, fat mass and fat free mass, while miRNA-30b was positively associated with infant weight, percent body fat content and fat mass at 1 month.

**Figure 4:**
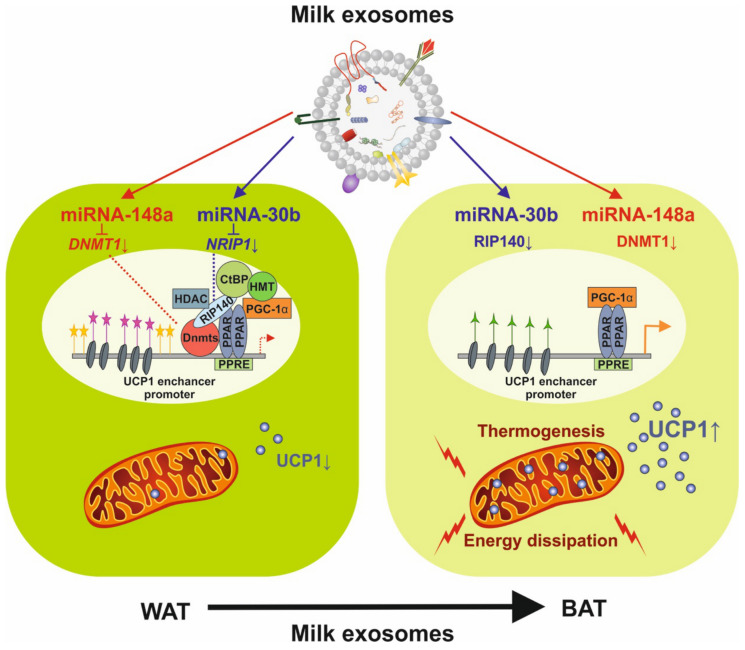
Potential impact of milk exosome-derived miRNA-148a and miRNA-30b on the expression of uncoupling protein 1 (UCP1). miRNA-148a inhibits DNA methyltransferase 1 (DNMT1) and miRNA-30b directly targets the mRNA of *NRIP1* encoding receptor-interacting protein 140 (RIP140), activating the enhancer/promoter of UCP1 and increasing UCP1 expression, which drives thermogenesis and the conversion of white adipose tissue (WAT) into brown adipose tissue (BAT).

There is translational evidence miRNA-148a and miRNA-30b control the expression of uncoupling protein 1 (UCP1) in BET mitochondria that are functionally thermogenic (Figure 4) [283,400,401]. It has been shown in murine adipose tissue that UCP1 expression is increased by *UCP1* enhancer methylation [402,403]. A previous study revealed that RIP140 and DNMT1 are both involved in the methylation of the enhancer and promoter of the murine *UCP1* gene [404]. In mouse adipocytes, RIP140 has been shown to elicit DNA methylation of the *UCP1* enhancer and promoter through binding to DNMT1, leading to transcriptional repression [404]. Tissue-dependent regulation of DNMT1 activity may be involved in the variation of DNA methylation of the *UCP1* enhancer and promoter [404]. MEX miRNA-148a-mediated suppression of DNMT1 may thus increase the expression of UCP1 enhancing thermogenesis and conversion of white to beige/brown adipocytes.

Xi et al. [405] reported that levels of miRNA-30b, let-7a and miRNA-378 in colostrum were negatively correlated with maternal pre-pregnancy BMI. Intriguingly, miRNA-30b/c concentrations are greatly increased during adipocyte differentiation and are stimulated by cold exposure or the β-adrenergic receptor activator. Over-expression and knockdown of miRNA-30b and miRNA-30c induced and suppressed the expression of thermogenic genes such as *UCP1* and death-inducing DFFA-like effector A (*CIDEA*) in brown adipocytes [244], respectively. Of note, the promoter activity of the lipid droplet protein CIDEA is repressed by RIP140 and induced by PGC-1α mediated through the binding of estrogen-related receptor-α (ERRα) and nuclear respiratory factor 1 (NRF-1) to their cognate binding sites [406]. RIP140 interacts directly with PGC-1α and suppresses its activity [406]. It is widely accepted that PGC-1α acts as a mediator of mitochondrial biogenesis [407,408].

Forced expression of miRNA-30b/c also significantly increased thermogenic gene expression and mitochondrial respiration in primary adipocytes derived from subcutaneous WAT, demonstrating a promoting effect of miRNAs on the development of BET. In addition, knockdown of miRNA-30b/c repressed UCP1 expression in BAT in vivo. Notably, miRNA-30b/c targets the 3′UTR region of the mRNA of RIP140 (*NRIP1*) [244]. Thus, over-expression of miRNA-30b/c significantly reduced RIP140 expression [244]. Mice devoid of RIP140 are lean, show resistance to high-fat diet-induced obesity and hepatic steatosis and have increased oxygen consumption. Although the process of adipogenesis is unaffected, expression of certain lipogenic enzymes is reduced. In contrast, genes involved in energy dissipation and mitochondrial uncoupling, including *UCP1*, are markedly increased [409]. Consistent with RIP140 as a target of miRNA-30b/c in regulating thermogenic gene expression, over-expression of RIP140 greatly suppressed the promoting effect of miRNA-30b/c on the expression of UCP1 and CIDEA in brown adipocytes [244]. RIP140 is a co-repressor for nuclear receptors that suppresses transcription from a broad program of metabolic genes and thereby controls energy homoeostasis in vivo [410,411]. Thus, miRNA-30b/c are key regulators of thermogenesis and uncover a new mechanism underlying the regulation of BAT function and the development of BET [244].

In contrast, over-expression of miRNA-155 in mice causes a reduction in BAT mass and impairment of BAT function [412]. miRNA-155 and its target, the adipogenic transcription factor CCAAT/enhancer-binding protein β (CEBP β), form a feedback loop integrating hormonal signals that regulate proliferation or differentiation [413].

Obviously, the appropriate balance of miRNA-148a, miRNA-30b/c and miRNA-155 controls the development of BET and BAT. MEX-derived miRNAs may contribute to the proper adjustment of miRNA-mediated adipogenic signaling. It is thus of critical concern that compared to HM, critical thermogenesis-regulating miRNAs are missing in infant formulas [42,164].

### 4.6. Milk Exosomes and Their Potential Impact on White Adipogenesis

There is increasing interest in the role of miRNAs in the regulation of BAT and BET as well as WAT [414]. However, studies on the impact of MEX on mesenchymal stem cell differentiation in humans and their role in the development of WAT, BET and BAT during the breastfeeding period are still missing. There is convincing evidence that miRNA-148a promotes the differentiation of pre-adipocytes to adipocytes [415,416,417,418,419,420]. Analysis of the upstream region of *MIR148A* locus identified a 3 kb region containing a functional cAMP-response element-binding protein (CREB) required for miRNA-148a expression in Ad-MSCs. The results suggest that miRNA-148a is a biomarker of obesity in human subjects and mouse models, which represents a CREB-modulated miRNA that acts to repress WNT1, thereby promoting adipocyte differentiation [415]. Furthermore, a potential X-box-binding protein 1 (XBP1) response element was found in the promoter region of *MIR148A*. An miRNA-148a mimic significantly restored adipogenic potential in XBP1-deficient 3T3-L1 cells providing evidence that XBP1s can suppress WNT10b by directly inducing miRNA-148a [417].

Type I procollagen mRNA expression is down-regulated during adipocyte differentiation [421]. Type 1 collagen inhibits adipogenic differentiation via Yes-associated protein (YAP) activation in vitro [422].

miRNA-148a-3p mimics have been shown to inhibit the expression of type I collagen in a model of alcoholic liver fibrosis [423], which may promote adipogenesis. In contrast, Reif et al. [27] demonstrated that HM-derived MEX significantly induced collagen type 1 expression in normal colon cells (CCD841).

It is generally accepted that CCAAT enhancer-binding protein-α (C/EBPα) and PPARγ are the key factors in modulating adipocyte differentiation and are the crucial genes for pre-adipocytes [412]. Recent evidence indicates that peroxisome proliferator-activated receptor-co-activator 1-β (PGC-1β) encoded on the *PGC1B* gene modulates the expression of key genes involved in adipogenesis during pre-adipocyte differentiation [424]. PGC-1β interference caused a significant decrease in lipid accumulation in chicken adipocytes with decreasing mRNA and protein abundances of PPARγ and sterol-regulatory element binding protein 1c (SREBP-1c), fatty acid synthase (FAS) and adipocyte type fatty acid binding protein (A-FABP) [424]. PGC-1β is restricted to the maintenance of basal mitochondrial function [408].

Kamei et al. [425] showed that PGC-1β functioned as a ligand for orphan ERRs. Transgenic mice over-expressing PGC-1β exhibited increased expression of medium-chain acyl-CoA dehydrogenase, an ERR target and a pivotal enzyme in mitochondrial β-oxidation in skeletal muscle. As a result, transgenic mice were hyperphagic, showed elevated energy expenditure and were resistant to obesity induced by a high-fat diet or by genetic abnormality. PGC-1β is an ERR protein ligand, whose expression induces a high-energy expenditure and antagonizes obesity. PGC-1β thus contributes to the control of energy balance [408,425]. Importantly, *PPARGC1B* is a direct target of miRNA-148a-3p. PGC-1β mRNAs were present at very low levels in 3T3-L1 pre-adipocytes and were markedly induced during adipocyte differentiation [425].

Over-expression of PGC-1β up-regulated the expressions of adipogenic and mitochondrial biosynthetic marker genes and promoted triacylglycerol accumulation during 3T3-L1 adipocyte differentiation. These observations suggest that PGC-1β modulates the expression of mitochondrial function and adipogenesis-related genes and affects white pre-adipocyte differentiation [426]. MEX-mediated transfer of miRNA148a via suppressing PGC-1β may thus interfere with the metabolic control of mitochondrial biogenesis and energy expenditure through the PGC-1 family regulatory network [427].

The miRNA-148a target *UCP3* is abundant in skeletal muscle and is involved in the regulation of postprandial thermogenesis [428]. The expression of UCP3 directly correlates to UCP1 abundance in BAT [429]. UCP3 abundance directly correlates with the degree of fatty acid β-oxidation in cell metabolism [430].

Cholecystokinin (CCK), which is secreted from endocrine IECs when the duodenum is filled with food, is a hypothalamic hormone that controls food intake [431]. CCK binds and signals via CCK1 receptor (CCK1R) and CCK2R. Notably, CCK2R knock out mice develop obesity associated with hyperphagia [432]. CCK2R deletion was associated with increased body weight and hypothalamic neuropeptide Y (NPY) content, which explains the increased food intake in CCK2R knockout mice [433]. Notably, the gene expressing CCK2R (*CCKBR*) is a direct target gene of miRNA-148a [434]. In addition, miRNA-148a attenuates the expression of LDLR and hepatic expression of ATP-binding cassette, subfamily A, member 1 (ABCA1) [435]. ABCA1 is a major regulator of plasma high density-lipoprotein (HDL) cholesterol responsible for the removal of excess cholesterol from peripheral cells and tissues [436,437]. Moreover, RIP140 negatively regulates the expression of ABCA1 by suppressing the expression and activity of liver X receptor (LXR) [438].

Apparently, MEX-derived miRNAs, especially the most abundant miRNA-148a of HM and MEX, have a significant impact on the regulation and programming of adipogenesis, energy, lipid and lipoprotein metabolism during the physiological period of breastfeeding.

### 4.7. Milk Exosomes and Bone Homeostasis

Mesenchymal stem cells (MSC) can differentiate into cells of the mesodermal lineage, such as adipocytes and osteocytes [439]. Postnatal bone development is characterized by substantial longitudinal bone growth and changes in skeletal size and shape. Bone is in a dynamic process of continuous remodeling, which helps to regulate calcium homeostasis, repair micro-damage to bones from everyday stress, and to shape the skeleton during growth [440]. During early childhood, both bone modelling (the formation and shaping of bone) and bone remodelling—the replacement or renewal of old bone—occur. The predominant process in childhood is bone modelling, while in adulthood, bone remodeling predominates [441]. Breastfeeding was beneficially associated with hip and total body areal bone mineral density (BMD) and total, cortical and trabecular volumetric BMD, as well as cortical thickness, porosity, trabecular number, separation and bone volume fraction at radius and/or tibia at 25 years of age in participants born prematurely, but there were no associations in those born at term [442]. During bone growth, bone-forming osteoblasts and bone-resorbing osteoclasts interact with blood vessel-forming endothelial cells [443].

There is compelling evidence that exosomes and their miRNA cargo play a crucial role in bone remodeling [444,445,446,447]. Notably, miRNA-148a-3p promotes adipocyte but inhibits osteoblast differentiation by targeting lysine-specific demethylase 6b [418]. MAF family members appear to play important roles in the regulation of MSC differentiation [448]. Nishikawa et al. [449] demonstrated that decreased expression of MAF in mouse MSCs, which regulated MSC bifurcation into osteoblasts and adipocytes, impaired osteogenesis. In fact, delayed bone formation has been observed in perinatal *Maf*^−/−^ mice [450]. Over-expression of miRNA-148a in CD14^+^ PBMCs promoted osteoclastogenesis, whereas inhibition of miRNA-148a attenuated osteoclastogenesis. MAFB is a transcription factor negatively regulating RANKL-induced osteoclastogenesis. miRNA-148a directly targets MAF and MAFB mRNA by binding to the 3′UTR and repressed MAFB protein expression [450].

There is recent interest in the impact of MEX and their miRNAs in osteogenesis and bone homeostasis. Intriguingly, Oliveira et al. [451] demonstrated that bovine MEX promoted osteoclast differentiation associated with an increased expression of c-Fos, which is important for the differentiation of pre-osteoclasts to osteoclasts [452]. The exposure of human MSCs to bovine MEX during 21 days resulted in less mineralization but higher cell proliferation and enhanced the expression of genes characteristic for immature osteoblasts [453]. Oral delivery of bovine MEX to female DBA1/J mice for 7 weeks increased osteoclast numbers but did not lead to more bone resorption [454]. In a model of bone loss induced by ovariectomy, increased osteoclast numbers in the femur were lowered by treatment with bovine MEX [454]. Yun et al. [455] recently reported that bovine colostrum-derived exosomes reduced osteoclast differentiation. The investigators induced osteoporosis in a mouse model using glucocorticoid pellets after orally administering colostrum exosomes for 2 months. Interestingly, the bone mineral density of colostrum exosome-fed mouse groups was significantly improved compared with the glucocorticoid-induced osteoporosis group without exosome treatment [455].

Recently, a potentially novel role for RIP140 in osteoclast differentiation, activity and bone turnover was reported [456]. RIP140 plays a physiological role in osteoclast precursors by regulating osteoclast differentiation through the formation of a suppressive transcription regulatory complex with testicular receptor 4 (TR4). RIP140 functions primarily by inhibiting osteoclast differentiation through forming a transcription-suppressor complex with TR4 to repress osteoclastogenic genes. These data reveal that monocyte/macrophage RIP140/TR4 complexes may serve as a critical transcription regulatory complex maintaining homeostasis of osteoclast differentiation, activity and coupling with osteoblast formation. MEX miRNA-30b mediated suppression of RIP140 may thus modify the balance of osteoclastogenesis towards osteoblastogenesis, which may be a meaningful mechanism for bone growth and bone modeling of the immobile newborn infant because bone formation of osteocytes physiologically requires mechanical stimuli [457].

Cortical bone development includes both pore closure and accumulation of high density bone. These processes require suppression of GP130-STAT3 signalling in osteocytes [458], which may be supported by MEX miRNA-148a-mediated suppression of GP130.

Taken together, translational evidence indicates that MEX under physiological conditions promote osteoclasts formation compared to osteoblasts during the postnatal period.

The potential impact of MEX miRNA-148a on adipocyte differentiation in relation to osteogenesis may secure energy reserves for the newborn. MEX-mediated promotion of osteoclast activity is of pivotal importance for the growing bone. In fact, osteoclasts not only resorb bone, but they also secrete anabolic signals that induce MSCs and osteoblasts to initiate osteogenesis in resorption lacuna (remodeling) or another non-resorbed site (modeling) [459].

Table 2 presents selected key targets of miRNA-148a, the predominant miRNA of MEX.

## 5. Milk Processing and Exosome Bioavailability

There is recent interest to use MEX and their miRNA cargo for the treatment and prevention of NEC [230] and to supplement MEX-deficient artificial formula [460]. The recovery of MEX after heat treatment depends on temperature and heat exposure time. Whereas UHT (135 °C, >1 s) and boiling (100 °C) of commercial cow milk destroys MEVs and MEX and their miRNA cargo [139,140], pasteurization (72–78 °C, >15 s) of commercial cow milk did not affect MEV numbers and preserved nearly 25–40% of milk’s total small RNAs [139]. HoP of HM (62.5 °C, 30 min) resulted in a significant decrease in MEX [22]. High pressure processing (HPP) of HM caused a statistically insignificant decrease in the number of miRNA reads compared to unprocessed material, whereas HoP led to a 302-fold decrease in exosomes not leaving enough reads for miRNA analysis [22]. It has been suggested that UV-C irradiation (UVC) is potentially a gentler method than HoP for pasteurizing donor milk for preterm infants preserving HM’s bioactive factors [461]. However, the effects of UV-C irradiation on MEX structure and bioavailbilty have not yet been studied. Other recent preservation methods of HM focus on freezing, lyophilization and freeze-drying [462,463,464,465]. These studies as well do not yet provide data on MEX and MEX miRNA bioavailability. Notably, lyophilization of exosomes without the cryoprotectant trehalose results in exosome aggregation, while the addition of trehalose prevents aggregation during lyophilization [466,467].

## 6. Conclusions

Human milk is a complex biological liquid comparable to blood that contains cells and multifaceted biological compounds including carrier systems, which provide nutrition to infants and help to develop their immune and metabolic systems [319,468]. The presence of secretory immunoglobulins (IgA), leukocytes, stem cells, lysozyme, lactoferrin, etc., in HM and their role in imparting passive immunity to infants, as well as modulating development of an infant’s immune system, is well-established. There is compelling evidence that MEX, a special subclass of HM’s large spectrum of MEVs, represent critical signalosomes that transfer regulatory RNAs, signaling proteins and mediator lipids that orchestrate epigenetic programming of the immune system and metabolism during a critical postnatal window of mammalian development [29,30,33,35,124,125].

Although the majority of studies presented here focused on biological information provided by MEX and their miRNAs, especially miRNA-148a, the whole spectrum of MEVs and their multiple RNAs contributes to milk’s functionality and complex signaling that requires further studies to appreciate the physiology and signaling capacity of milk, the masterpiece of mammalian evolution.

Obviously, MEVs and MEX relay biological “big data” originating from the highly conserved lactation genome from the mother to her infant. In a highly responsive manner, MEX quantities and composition vary depending on the time of delivery (preterm/term), the course of lactation (colostrum, mature milk), environmental factors (maternal obesity, allergic sensitization) and hormonal factors (oxytocin, prolactin and melatonin). MEX stabilize IEC proliferation maturation and ISC activity and improve intestinal barrier function, including the formation of TJs, the mucus barrier and anti-microbial barrier. In addition, MEX and their miRNA cargo attenuate local intestinal inflammation, underlining their most favorable impact for the prevention and treatment of NEC. Apart from the beneficial effects of MEX on the intestine, we are beginning to appreciate their potential systemic interactions with the thymus, brain, liver, pancreatic β-cells, adipose tissues and bones during the restricted life period of breastfeeding.

Starting out with an only rudimentary scientific knowledge of milk, milk’s complex biology and functionality was misconceived by pediatrics of the 1920s and has been erroneously over-simplified as “just food”, allowing the large-scale introduction of unsweetened evaporated milk for the preparation of infant feeding formulas [469]. The artificial replacement of breastfeeding was promoted by rigorous marketing of formula as “the better milk for babies” [470]. After years of laborious adjustments of formula protein overload [471,472,473] with adipogenic mTORC1-activating amino acids [474,475], we are now facing another formula deficit, i.e., the absence of MEVs, MEX and MEX-delivered miRNAs [42,164]. The highly dynamic and complex biosynthesis and signaling of MEX and their miRNAs, including their incompletely understood lncRNAs and circRNAs, make a “static” formulation with selected miRNAs a bold venture. We should appreciate all signaling effectors of HM developed by millions of years of evolution of mammalian lactation [476]. The enrichment of MEX in all mammals and their highly conserved nucleotide sequence homology of the major MEX miRNAs underlines that MEX play a prominent evolutionary role during a most critical epigenetic and metabolic window of mammalian development. The potential impact of MEX and their cargo on global epigenetic and metabolic regulators such as DNMT1 and RIP140 exemplifies the complexity of this evolutionary system which is impossible to copy. It is incomprehensible that we expose our offspring to insufficiently controlled artificial feeding systems that we have not yet understood in detail.

The only secure way to guarantee optimized epigenetic and metabolic programming during the postnatal period is a strict return to the belief into the confidence and functionality of our own lactation genome. A normal BMI during pregnancy and avoidance of unnecessary Casarean sections and formula feeding but sufficient breastfeeding/programming may offer a great chance for the prevention of noncommunicable diseases of civilization, which appear to be communicable by a better understanding of HM´s postnatal imprinting mechansisms. For mothers unable to provide adequate breastfeeding, human donor milk may be a substitute but may already lead to deviations in epigenetic programming, as recently shown in a murine model [31].

At present, the processing and conservation methods of human donor milk have not been sufficiently controlled for the bioavailability of MEX and their complex cargo. The presented epigenetic impact of MEX miRNA signaling among other complex milk-derived signals allows the conclusion that babies are not simply “breast-fed” but are most importanly “programmed by the breast”, the charateristic gland defining mammals.

In 1913, Sir Truby King, a prominent child health reformer and proponent of breastfeeding, stated that breastfeeding is not only the best for the mother and her baby, but is a baby’s birthright [477]. With our contemporary knowledge of milk’s sophisticated and still mysterious molecular biology, we recommend that breastfeeding is best for infant health and development [478], offering a great chance for prevention of diseases of civilization [4].

## Figures and Tables

**Table 1:** Biological effects of milk-derived exosomes (MEX) in colitis and NEC models.

Model	MEX Source	Insulting Agents	Biological Effects	References
IEC-6 cells	Human	H_2_O_2_	Increased cell viability; protection from oxidative stress	[231]
IEC-6	Yak, Cow	Hypoxia	Yak-MEX increased survival of IEC-6 cells compared with bovine-MEX; yak-MEX promote oxygen-sensitive prolyl hydroxylase (PHD)-1 expression and decrease HIF-α, VEGF and p53	[72]
IEC	Porcine	LPS	Decreased LPS-induced TLR4/NF-κB signaling pathway activation; reduced LPS-induced apoptosis via the p53 pathway	[162]
IEC murine intestine	Porcine	Deoxynivalenol	Up-regulation of miRNA-181a, miRNA-30c, miRNA-365-5p and miRNA-769-3p in IPEC-J2 cells; suppression of p53 pathway; increased proliferation and TJs; inhibition of apoptosis	[85]
Premature Sprague–Dawley rat pups; IEC-6 cells	Human	Asphyxia, hypothermia, hypercaloric feed, hypoxia	Decrease in histological NEC grade; increased IEC cell proliferation; decreased apoptosis of IEC	[232]
Prominin-1^+^ ISCs of small intestines of neonatal rat	Human	H_2_O_2_	Increase in ISC viability; increased expression of LRG5, axin2, c-myc, cyclin D1, HES1, DII1, DII4	[77]
LS174T human colonic cells; C57BL/6 mice	Bovine	Hypoxia, hyperosmolar formula, LPS	Increased goblet cell numbers and mucin production; Increased expression trefoil factor 3 (TFF3) and mucin 2 (MUC2). Enhanced the expression of glucose-regulated protein 94 (GRP94)	[47]
C57BL/6J mice	Bovine	Dextran sulfate sodium	Decreased inflammation through the down-regulation of colitis-associated miRNAs, especially miRNA-125b, associated with a higher expression of the NF-κB inhibitor TNFAIP3	[84]
Newborn Sprague–Dawley rat pups; human intestinal epithelial FHC	Human, term/preterm	Hypoxia formula	Preterm MEX significantly enhanced proliferation and migration of IECs compared with term MEX	[233]
Intestinal organoids; C57BL/6 mice pups	Human	LPS	Decreased expression of TNF-α and TLR4	[163]
Balb/c mice	Human	Dextran sulfate sodium	MEX attenuated the severity of colitis induced by DSS and statistically reduced the histopathological scoring grade and shortening of the colon; reduced expression of IL-6, TNF-α, DNMT1 and DNMT3; up-regulation of TGF-β	[148]
*Mdr1a*^−/−^ mice (5 weeks old)	Bovine	60% MEX-deficient diet	Higher degree of intestinal lesions; deficiency of miRNA-200a-3p targeting Cxcl9 mRNA	[234]
Intestine of kindlin 2 knockout mice	Bovine	Kindlin 2 knockout	Decrease in macroscopic colitis score in MEX-treated mice compared with untreated mice	[235]
Intestinal organoids of C57BL/6 mouse pups	Human	Hypoxia formula, LPS	Decreased IL-6 mRNA expression; decreased injury score and MPO activity; increase in goblet cell number and MUC2 mRNA expression	[92]

**Table 2:** Selected targets of MEX-derived miRNA-148a-3p and functional outcomes.

miRNA-148a 3p Target Genes	Potential Functional Outcomes During Breastfeeding
*PRKAA1*	Inhibition of AMPK; suppression of pancreatic β-cell activation; increased IEC-and β-cell mTORC1 activity with IEC and β-cell proliferation
*PRKAG2*	Inhibition of AMPK; suppression of β-cell activation; increased IEC and β-cell mTORC1 activity with IEC- and βcell proliferation
*PPARGC1B*	Inhibition of PCG-1β; Reduced mitochondrial function
*UCP3*	Reduced fatty acid β-oxidation and energy expenditure
*CCK2R*	Reduced satiety signals increasing milk/food intake
*MAFB*	Increased osteoclastogenesis
*LDLR*	Reduced hepatic LDL cholesterol uptake
*ABCA1*	Reduced HDL-mediated reverse cholesterol transport
*COL1A1*	Reduced collagen I synthesis
*IL6ST (GP130)*	Reduced expression of GP130 resulting in attenuated IL-6 signaling, increased cortical bone maturation
*IKBKA*	Inhibition of IκB kinase α and NF-κB signaling, suppression of inflammation
*IKBKB*	Inhibition of IκB kinase β and NF-κB signaling, suppression of inflammation
*CAMK2A*	Inhibition of calcium/calmodulin-dependent protein kinase IIα and downstream TLR4 signaling
*DNMT1*	Inhibition of DNA methyltransferase 1 increasing epigenetic expression of developmental genes (*INS*, *IGF1*; *SNCA*, *FOXP3*) and suppression of RIP140 expression and RIP140-dependent nuclear receptors and transcription factors such as PGC-1α

## Data Availability

All data are derived from the PubMed database and are in agreement with MDPI Research Data Policies.

## List of Abbreviations (in Alphabetical Order)

AD	atopic dermatitis
AMPK	adenosine monophosphate-activated protein kinase
α-syn	α-synuclein
BAT	brown adipose tissue
BCAA	branched-chain amino acid
BCKD	branched-chain α-ketoacid dehydrogenase
BET	beige adipose tissue
BLG	β-lactoglobulin
BMI	body mass index
BMMF	bovine meat and milk factor
CAV3	caveolin 3
CaMKIIα	calcium/calmodulin-dependent protein kinase IIα
CARMA1	CARD-containing MAGUK protein 1
CCK	cholecystokinin
CCKR	cholecystokinin receptor
CCR	chemokine, CC motif, receptor
CDX2	caudal-type homeobox transcription factor 2
CEBP	CCAAT/enhancer-binding protein
CIDEA	death-inducing DFFA-like effector A
circRNA	circular RNA
CLDN1	claudin 1
CMA	cow milk alllergy
CRC	colorectal cancer
CYR61	cystein-rich protein 61
DBT	dihydrolipoamide branched-chain transacylase
DC	dendritic cell
DMBT1	deleted in malignant brain tumors 1
DNMT1	DNA methyltransferase 1
DON	deoxynivalenol
DSS	dextran sodium sulfate
ERRα	estrogen-related receptor-α
ER	endoplasmic reticulum
EV	extracellular vesicle
FcRn	neonatal crystallizable fragment receptor
FOXP3	forkhead box transcription factor P3
GATA4	GATA-binding protein 4
GI	gastrointestinal tract
GP130	interleukin 6 signal transducer
GRP94	glucose-regulated protein 94
GSIS	glucose-stimulated insulin secretion
GSK3	glycogen synthase kinase 3
HM	human milk
HDAC4	histone deacetylase 4
hMSC	human mesenchymal stem cell
HoP	Holder pasteurization
HSP	heat shock protein
IBD	inflammatory bowel disease
IEC	intestinal epithelial cell
IFN	interferon
IgA	immunoglobulin A
IgG	immunoglobulin G
IGF-1	insulin-like growth factor 1
IGF1R	IGF-1 receptor
IκB	inhibitor of κB
IKK	IκB kinase
IL-1	interleukin 1
IL-2	interleukin 2
IL-6	interleukin 6
IL-8	interleukin 8
IL-10	interleukin 10
IL-17	interleukin 17
ISC	intestinal stem cell
iTreg	inducible regulatory T cell
LDLR	low density-lipoprotein receptor
LGR5	leucine-rich-repeat-containing G-protein-coupled receptor 5
lncRNA	long non-coding RNA
LRP6	low-density lipoprotein receptor-related protein 6
MesD	mesoderm development
MEV	milk extracellular vesicle
MEX	milk exosome
MFG	milk fat globule
MFG-E8	MFG epidermal growth factor 8
MGEC	mammary gland epithelial cell
MHC	major histocompatibility complex
miRNA	micro-ribonucleic acid
MPO	myeloperoxidase
mTORC1	mechanistic target of rapamycin complex 1
MUC1 MUC2	mucin 1 mucin 2
MyD88	MyD88 innate immune signal transduction adaptor
NCOA1	nuclear receptor co-activator 1
NEC	necrotizing enterocolitis
NF-κB	RELA protooncogene, NFKB subunit
NK	natural killer
OCLN	occludin
OTU	operational taxonomic unit
PBMC	peripheral blood mononuclear cell
PCNA	proliferating cell nuclear antigen
PGC-1α	peroxisome proliferator-activated receptor-γ co-activator 1α
PGC-1β	peroxisome proliferator-activated receptor-γ co-activator 1β
PI3K	phosphatidylinositol 3-kinase
PPAR	peroxisome proliferator-activated receptor
PPARGC1A	peroxisome proliferator-activated receptor-γ co-activator-1α (PGC-1α)
PRKAA1	catalytic subunit α1 of AMPK
PRKAG2	regulatory subunit γ2 of AMPK
PTEN	phosphatase and tensin homolog
PURB	purine-rich element binding protein B
RIP140	receptor interacting protein 140
RegIIIγ	regenerating islet-derived 3γ
ROR	RAR-related orphan receptor
SIRT1	sirtuin 1
SNARE	soluble N-ethylmaleimide-sensitive factor attachment protein receptor
SOCS1	signal transducer and activator of transcription 1
SNCA	α-synuclein
STAT	signal transducer and activator of transcription
TCR	T cell receptor
TGFβ	transforming growth factor-β
TJ	tight junction
TLR	toll-like receptor
TNFα	tumor necrosis factor-α
TNFAIP3	TNFα-induced protein 3
TFF3	trefoil factor 3
T2DM	type 2 diabetes mellitus
Treg	FOXP3+ regulatory T cell
TSDR	Treg-specific demethylated region
TSG101	tumor susceptibility gene 101
UCP1	uncoupling protein 1
UCP2	uncoupling protein 2
UCP3	uncoupling protein 3
UHT	ultraheat-treated
3′UTR	3′-untranslated region
VEGF	vascular endothelial growth factor
WAT	white adipose tissue
WNT	wingless
ZO-1	zonula occludens 1
